# Chemometric-Guided Approaches for Profiling and Authenticating Botanical Materials

**DOI:** 10.3389/fnut.2021.780228

**Published:** 2021-11-26

**Authors:** Evelyn J. Abraham, Joshua J. Kellogg

**Affiliations:** ^1^Intercollege Graduate Degree Program in Plant Biology, The Pennsylvania State University (PSU), University Park, PA, United States; ^2^Department of Veterinary and Biomedical Sciences, The Pennsylvania State University, University Park, PA, United States

**Keywords:** metabolomics, adulteration, multi-omics, dietary supplements, biochemometrics, chemometrics, botanicals, authentication

## Abstract

Botanical supplements with broad traditional and medicinal uses represent an area of growing importance for American health management; 25% of U.S. adults use dietary supplements daily and collectively spent over $9. 5 billion in 2019 in herbal and botanical supplements alone. To understand how natural products benefit human health and determine potential safety concerns, careful *in vitro, in vivo*, and clinical studies are required. However, botanicals are innately complex systems, with complicated compositions that defy many standard analytical approaches and fluctuate based upon a plethora of factors, including genetics, growth conditions, and harvesting/processing procedures. Robust studies rely upon accurate identification of the plant material, and botanicals' increasing economic and health importance demand reproducible sourcing, as well as assessment of contamination or adulteration. These quality control needs for botanical products remain a significant problem plaguing researchers in academia as well as the supplement industry, thus posing a risk to consumers and possibly rendering clinical data irreproducible and/or irrelevant. Chemometric approaches that analyze the small molecule composition of materials provide a reliable and high-throughput avenue for botanical authentication. This review emphasizes the need for consistent material and provides insight into the roles of various modern chemometric analyses in evaluating and authenticating botanicals, focusing on advanced methodologies, including targeted and untargeted metabolite analysis, as well as the role of multivariate statistical modeling and machine learning in phytochemical characterization. Furthermore, we will discuss how chemometric approaches can be integrated with orthogonal techniques to provide a more robust approach to authentication, and provide directions for future research.

## Introduction

Botanical medicines and dietary supplements represent a growing facet of personal health and medical care for Americans; the 2017 survey from the Council for Responsible Nutrition found that botanicals make up ca. 39% of total dietary supplement usage for adults in the United States (1), and US sales of herbal supplements totaled $9.6 billion in 2019, an annual increase of 8.6% (2). The use of botanical medicines and dietary supplements has come to include patients receiving disease therapy, such as cancer (3) and chronic obstructive pulmonary disease (COPD) (4). The increase in economic and biomedical relevance of botanicals have led to a rise in research interest surrounding their potential health benefits, including support from the National Institutes of Health (5). The US National Library of Medicine's clinical trial tracker (clinicaltrials.gov) had >140 active clinical trials involving “herbal” or “botanical” preparations listed (accessed July 30, 2021) (6). However, the veracity of biomedical research, whether it is *in vitro* studies or clinical trials, is predicated on the authenticity and purity of the botanical(s) being studied. Botanical products are inherently complex chemical mixtures that can vary depending on abiotic and biotic factors during growth and post-harvest processing. Complicating this is the fact that products can be obtained from multiple producers and growers, potentially with multiple sources of raw material and processing techniques. Thus, to ensure the authenticity, efficacy, and safety of botanical dietary supplements, complex multi-faceted methods are required. This review focuses on chemometric and orthogonal methods for profiling, analyzing, and comparing botanical systems. We first provide opportunities and limitations of traditional botanical product authentication, followed by an overview of alternative chemometric approaches, then delve into a plethora of multivariate statistical approaches for botanical evaluation and present a workflow for how researchers can rationally select an analytical model based on data types and goals.

## Opportunities and Limitations of Traditional Approaches

### Morphology

Plant morphology is the traditional approach to botanical product authentication, based on leaf shape and size and arrangement, color, life cycle changes, and other phenotypic factors. The combination of modern resources for plant identification and expansive collections of medicinal plant herbarium vouchers allows for fairly accurate morphological characterization (7, 8). Although trained specialists provide the most accurate identifications, guidebooks and phone applications provide a simple, inexpensive avenue for authentication. Increased accuracy results from micromorphology which allows species-specific evaluations of pollen shape, pore size, and other microscopic traits (9, 10). Recently, machine learning and image processing software have led to high-thruput identification of medicinal plants based on predefined characteristics and extensive training datasets (11, 12).

Despite its strengths, morphology-based identification is limited and often impractical, especially for rare plants. Similar environments and evolution pathways can result in unrelated plants with strong morphological resemblances but differing medicinal properties. Furthermore, important morphological information is lost when plants are dried or powdered, such as leaf shape and texture. Morphology also varies between plant parts, and recorded information for identifying plants based on below ground parts rarely exists. While certain root characteristics are useful, such as stone cells, auxiliary root angle, and rhizome length, the literature for species level identification is lacking and often contradictory between labs (13–16). Taxonomic identification is further complicated by vernacular names, which vary based on culture, location, language, and subspecies (17, 18).

### Genetics

Genetic approaches, namely DNA barcoding and genome sequencing, are powerful tools for herbal product authentication. DNA can be extracted from fresh or dried tissue and is often effective with post-processed material (19). Primer-based methods are the most straightforward approach to DNA based identification: predefined primers for single genes (ITS2), a combination of genes (*matK* and *rbcL*), or chloroplast genomes (18, 20–23) amply specific fragments know to vary between species and have potential to differentiate morphologically and genetically similar species (24). Extensive sequence libraries exist which simplifies species identification; rare and understudied species are not thoroughly represented though (24). As sequencing becomes increasingly advanced and affordable, the applicability of genetic marker-based identification of a broad range of botanicals will increase.

DNA barcoding, including random amplification of polymorphic DNA (RAPD) (25) and inter-simple sequence repeats (ISSR) (26), provides a robust evaluation of genome diversity through examination of the presence/absence of more than 20 random fragments of polymorphic DNA at a time. Primer-based approaches amplify random segments of DNA to compare polymorphic variations among species. Although DNA barcoding is reliable, it is time consuming and requires meticulous method optimization for each application. Further, there is low resolution at the species or sub-species level (27). Recent advances in metabarcoding, which combines next-generation sequencing with bioinformatics, has greatly improved the ability to detect adulteration and supplementation in herbal products (28–30). Notably, the EU and other governing bodies suggest metabarcoding to evaluate the identity and safety of botanical products (31). For example, Seethapathy et al. used metabarcoding to determine that over 24% of Ayurvedic herbal products tested do not contain the botanical as labeled (28). However, metabarcoding is expensive and requires a reference DNA library and pre-defined genetic markers. So, for rare species or those without sequenced genomes, metabarcoding is ineffective as a quality control approach (32).

While genetic approaches have proven useful for botanical product quality control, there are limitations. Plant tissue is damaged and degraded during processing procedures, hindering extractions of high-quality DNA (33). Since genetic approaches do not provide quantitative data, there is limited ability to determine relative abundances of different species within a product. Thus, DNA barcoding does not allow trace contamination, as from shared equipment, to be discerned from intentional, large-scale adulteration of products. A final limitation is the inability to evaluate medicinal properties through barcoding based approaches. The medicinal value of a product is largely based on its chemical constituents. Without detailed chemical analysis, the presence and relative abundance of specific medicinal compounds is unknown. So, while genetics may be able to detect adulteration, it cannot determine a products actual medicinal value. Thus, chemical evaluation serves to both authenticate botanicals and provide information of a product's bioactive potential.

## Targeted Analysis of Biomarkers

A simple and common approach to herbal product quality control is the use of small-molecule based targeted analysis. This approach uses individual and small groups of compounds specific to the botanical in question. Using a targeted analysis allows quick verification the product contains the plants as advertised. This section outlines the targeted analysis workflow, with examples and explanations of the pros and cons of targeted approaches.

### Single Biomarker Approach

The first step in using small molecule chemistry to serve as biomarkers of quality and authenticity is to identify a targeted metabolite or small set of metabolites specific to the botanical in question. Since many commercially available botanical medicines and dietary supplements are fairly well characterized in scientific literature, the identification of predominant metabolites (also known as ‘marker compounds') is fairly straightforward. These targeted compounds are analyzed by a chemical methodology and compared against reference standards and literature values; common analytical techniques include charged aerosol detection (CAD), ultraviolet-visible (UV/VIS) spectrophotometry, and mass spectrometry (MS), often with chromatographic separation beforehand (liquid chromatography, “LC”, or gas chromatography, “GC” being the two primary forms). Nuclear magnetic resonance (NMR) is an analytical technique that has become more quantitative recently (qNMR) to facilitate comparisons between complex botanical samples (34–36).

However, axiomatic to using a defined marker compound is the knowledge of the chemistry of the system at hand and the commercial availability (or the ability to isolate and conclusively identify) of the target marker compounds. While many botanicals on the market have well-developed chemical libraries and/or have monographs detailing their chemical composition [including the German Commission E (37), US Pharmacopeia (38), and Tyler's Herbs of Choice (39)], not every botanical, nor every potential dietary supplement, is as thoroughly studied, and gaps in the literature of even well-known botanicals still exist today. The choice of marker compound also should, but doesn't necessarily, have relevance to the putative biological activity of the botanical medicine or dietary supplement. Finally, standards must be available to construct calibration curves; if they are not commercially available, researchers face the daunting task of isolating and elucidating the structure prior use as a marker compound (40).

Furthermore, tying authenticity to a single compound overlooks the broader chemical landscape present in the botanical product, and can leave products susceptible to potential adulteration. Single-point analyses can be confounded by spiking with specific compounds or mixtures that might bypass quality control procedures. One example is the discovery by Chandra et al. of adulteration in ginkgo (*Gingko biloba*) extracts spiked with either single isolated flavonoids or flavonoid-rich mixtures (41). As the broad category “flavone glycosides” was chosen by the gingko market as an authenticity marker, it was prone to spiking by flavones (e.g., quercetin, kaempferol, and isorhamnetin) to meet the quality criteria. In fact, three out of eight products analyzed in the study that were labeled to contain ginkgo extracts actually resembled those of commercial extracts from Japanese sophora (*Styphnolobium japonicum*) (41). In other cases, botanical dietary supplements have been doped with dyes or other synthetic mixtures to deceive single molecule quality control methods (42). Supplements with alleged weight loss properties were spiked with alkaloid derivatives, ephedra stimulants, or androgenic steroids (43, 44). Spiking and adulteration can also be used to bypass negative controls searching for known contaminants/adulterants; 1,3-dimethylamylamine (1,3-DMAA) is one case study. The United States Food and Drug Administration (FDA) had banned 1,3-DMAA in 2016 and pulled all products containing the stimulant from shelves because of an increased incidence of ER visits correlated with this stimulant, as well as failure to meet regulatory conditions (45). However, investigations by Cohen et al. revealed 1,3-DMAA analogs present in multiple weight loss supplements (five out of six tested), illustrating how adulteration can be used to sidestep regulatory authorities with potentially toxic constituents (45).

### Molecular “Fingerprints”

Beyond single molecules for targeted biomarker detection, researchers can collect information on a range of molecules or a “chemical fingerprint” that exemplifies a more robust and nuanced representation of the botanical's metabolite profile. Using multiple components blunts the potential for metabolite spiking (as seen with single marker compound approaches) and can provide more selective and sensitive analysis for distinguishing authentic material. Lv et al. (2016) developed an HPLC-based fingerprint to differentiate species and geographical origins of *Rhizoma coptidis* using six distinct alkaloids (46), while eight organic acids were used to distinguish between *Castanea* spp. Buds (47), and Parveen et al. validated an UHPLC-UV-MS method incorporating 10 standard compounds to distinguish closely related *Tinospora* species (48). Even AOAC's official method for some botanicals incorporates multiple compounds; their method 2015.007 for investigating Ashwagandha (*Withania somnifera*) employs 10 withanolide glycosides and aglycones (49). However, multi-molecular chemical “fingerprints” are more time- and labor-intensive approaches, as they require the quantitation of multiple compounds with different linear ranges and limits of detection and quantitation (LOD and LOQ, respectively). This also does not circumvent the issue with single biomarker approaches needing reliable, commercially available standards in order to determine the overall fingerprint and quantitation for the analysis.

## Metabolomics

The ‘metabolome' is generally defined as the complete set of small molecules produced by an organism or biological sample at any given point in time. Metabolomics, therefore, is the unbiased, holistic measurement of the metabolome (though practically speaking there is no single analytical approach capable of measuring all small molecules in one experiment), and the relative areas or heights of signals within the metabolome can be employed as a basis for comparison between two or more samples. As such, metabolomics provides a powerful tool for understanding the complete chemical makeup of an herbal product, which can be used for efficient and accurate quality control and authentication. Metabolomics characterizes the chemical relationships that underlie variations based upon genotype, origin (50), climate (51), or other biotic or abiotic interactions (52–54). While a variety of analytical inputs can be used to generate metabolome data – including Fourier-Transformed infrared spectroscopy (FT-IR), charged aerosol detection (CAD), ultraviolet-visible (UV/VIS) spectrophotometry, mass spectrometry (MS), and Nuclear Magnetic Resonance (NMR) spectroscopy – the two primary analytical approaches employed for the majority of metabolomics studies are liquid chromatography coupled to mass spectrometry (LC-MS) and NMR spectroscopy. These two provide incredible sensitivity and selectivity in profiling a large fraction of the metabolome of a sample, while also offering detailed structural information crucial for metabolite annotation for the authentication of botanical dietary supplements and medicines (55, 56). The advances of metabolomics techniques is not the focus of this review, the incredible innovation and progress that has been achieved in metabolomics experiments have been discussed elsewhere (57–59).

As relative comparisons are being made across a large dataset (often hundreds to thousands of peaks in a single metabolome data matrix), the chemical identification of the peaks is not necessary at the outset of the experiment and analysis. Thus, untargeted metabolomics studies can compare complex samples with no a priori knowledge of their constituents (60) and do not require the acquisition of analytical standards to complete comparative analyses, a distinct advantage over the targeted or fingerprinting approaches described above.

## Chemometric Approaches for Pattern Recognition and Similarity Determination

**Figure 1:**
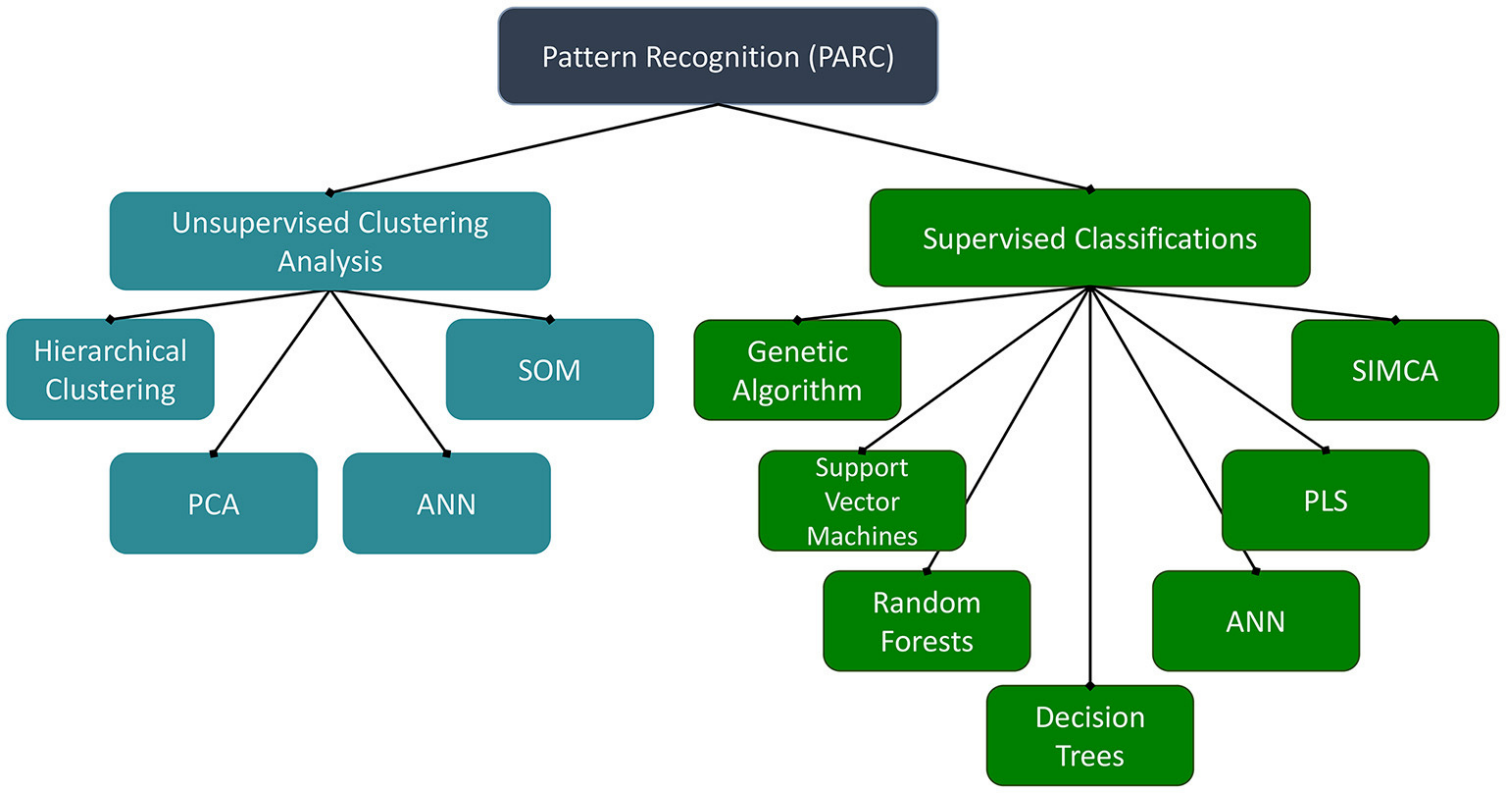
Pattern recognition methods. ANN, artificial neural networks; PCA, principal component analysis; PLS, partial least squares; SIMCA, soft independent modeling of class analogy; SOM, self-organizing maps.

While a valuable tool for authentication of herbal products, the innate complexity of metabolomic datasets can be daunting when developing novel quality control approaches. One of the major challenges facing metabolomic (or other molecular fingerprinting approaches) is not the collection of the data, but instead the processing, analysis, and interpretation of the expansive datasets that are often generated. In metabolomics, the data matrices often have more columns (independent variables, such as *m/z*-retention time pairs or NMR signal buckets) than rows (samples) and are known as “landscape” matrices. “Chemometrics” refers to the application of statistical methods to discover relevant analysis and maximize the information obtained from the chemical datasets (61). For the authentication of botanical materials, chemometric pattern recognition approaches are the most prevalent. There are a variety of multivariate mathematical–statistical methods for prediction and pattern recognition (Figure 1), which have disparate criteria for successful application to complex chemical datasets.

### Data Preparation for Chemometric Analysis

In any statistical analysis, the robustness of the predictions and inference is limited by the quality of the data that is input into the model. For chemometric analysis, there are a number of aspects of the dataset that will contribute to the overall quality and reliability of the resulting model. One aspect of note is the reproducibility of analytical data. Variations in extraction protocol, sample handling as well as the mass spectrometer detection itself (mass analyzer, detector, and even the chromatography components) preclude facile comparisons between labs. This can potentially lead to differing raw spectral data, as well as variations in results obtained (62).

Raw spectral data, from any analytical source (LC-MS, GC-MS, NMR, FT-IR, etc.) must be processed in order for the statistics to be effective. For some spectral data (e.g., ^1^H-NMR and FT-IR), the data is traditionally sliced into “bins” that are then used as individual features in the dataset (63, 64). Mass spectrometry data is obtained as discreet features (unique *m/z*-retention time pairs), yet still requires multi-step “preprocessing” to identify peaks and align the data. There are numerous methods and workflows to preprocess spectral data, and have been examined and reviewed exhaustively elsewhere (65–71). While most open access preprocessing software yields similar performance in detection of actual peaks (“true” features) from the data [as examined by Li et al. (68)], the abundance of parameters needed to fine tune in order to develop a robust final dataset can be challenging for researchers. The subsequent scaling, centering, and normalization of the dataset can also play a factor in the resulting statistical analysis (72, 73). Thus, careful treatment of the raw data during preprocessing is critical to downstream chemometric analyses in order to obtain reproducible and reliable interpretations of the data. The potential for variations in the processing of the data is a persuasive argument in favor of the trend in metabolomics to encourage open science by depositing the spectral data, as well as metadata associated with the preprocessing parameters used, in accordance with the FAIR (Findable, Accessible, Interoperable, and Reusable) data principles (74).

### Unsupervised Approaches

Unsupervised methods are the (relatively) simplest ways of classifying large chemical datasets, designed to analyze data that can only be arranged in one matrix. These methods are “unsupervised” in the sense that no data classifications are known before the analysis; instead data structures are revealed through these pattern recognition methods. Researches should be aware of the differences between hard and soft classification techniques.

#### Principal Component Analysis (PCA)

**Figure 2:**
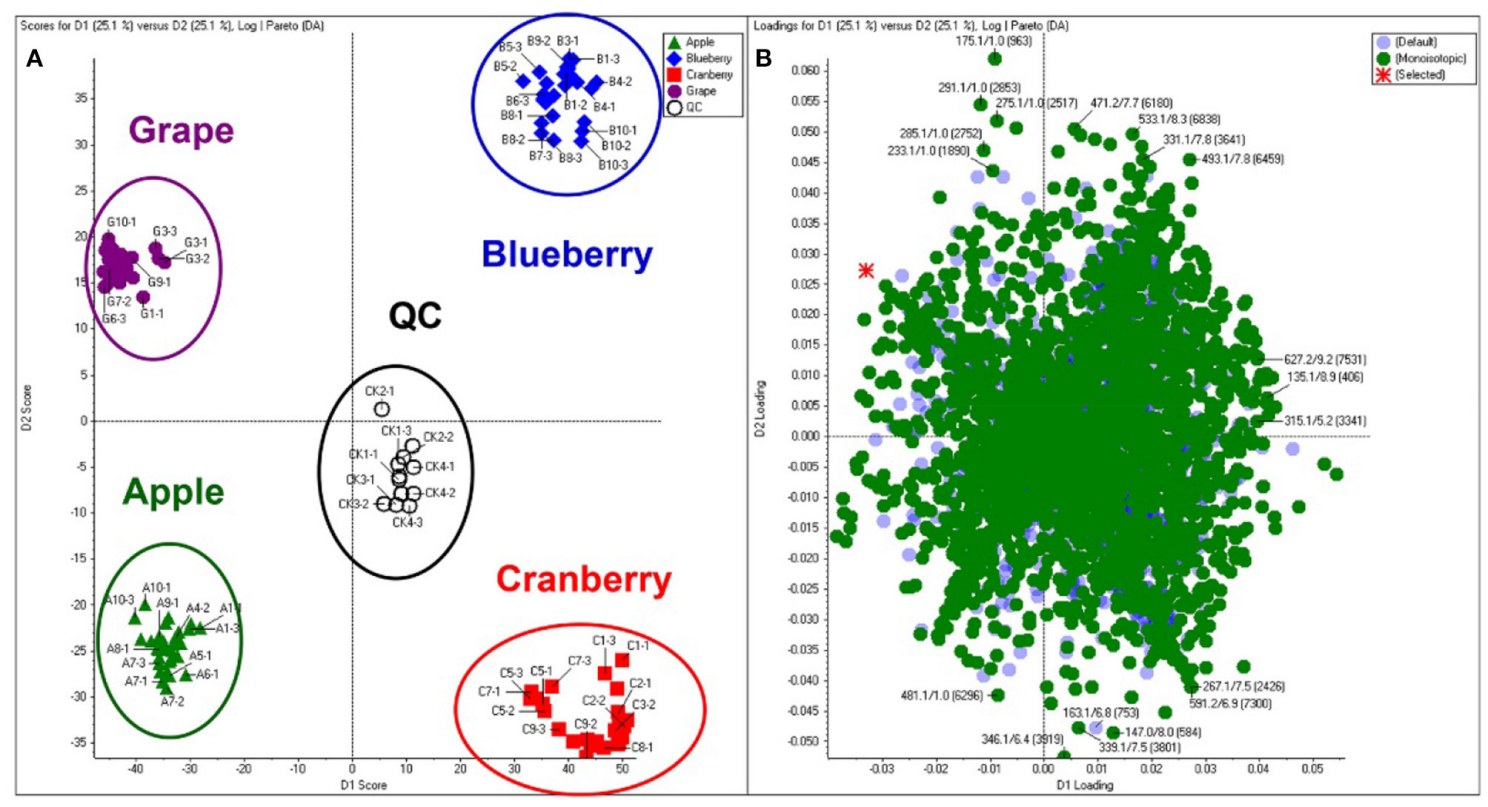
Principal component analysis (PCA) scores **(A)** and loadings **(B)** plot demonstrating differentiation between fruit juices based upon untargeted metabolomic analysis. Reproduced with permission from Zhang et al. (76). Copyright 2018, American Chemical Society.

**Figure 3:**
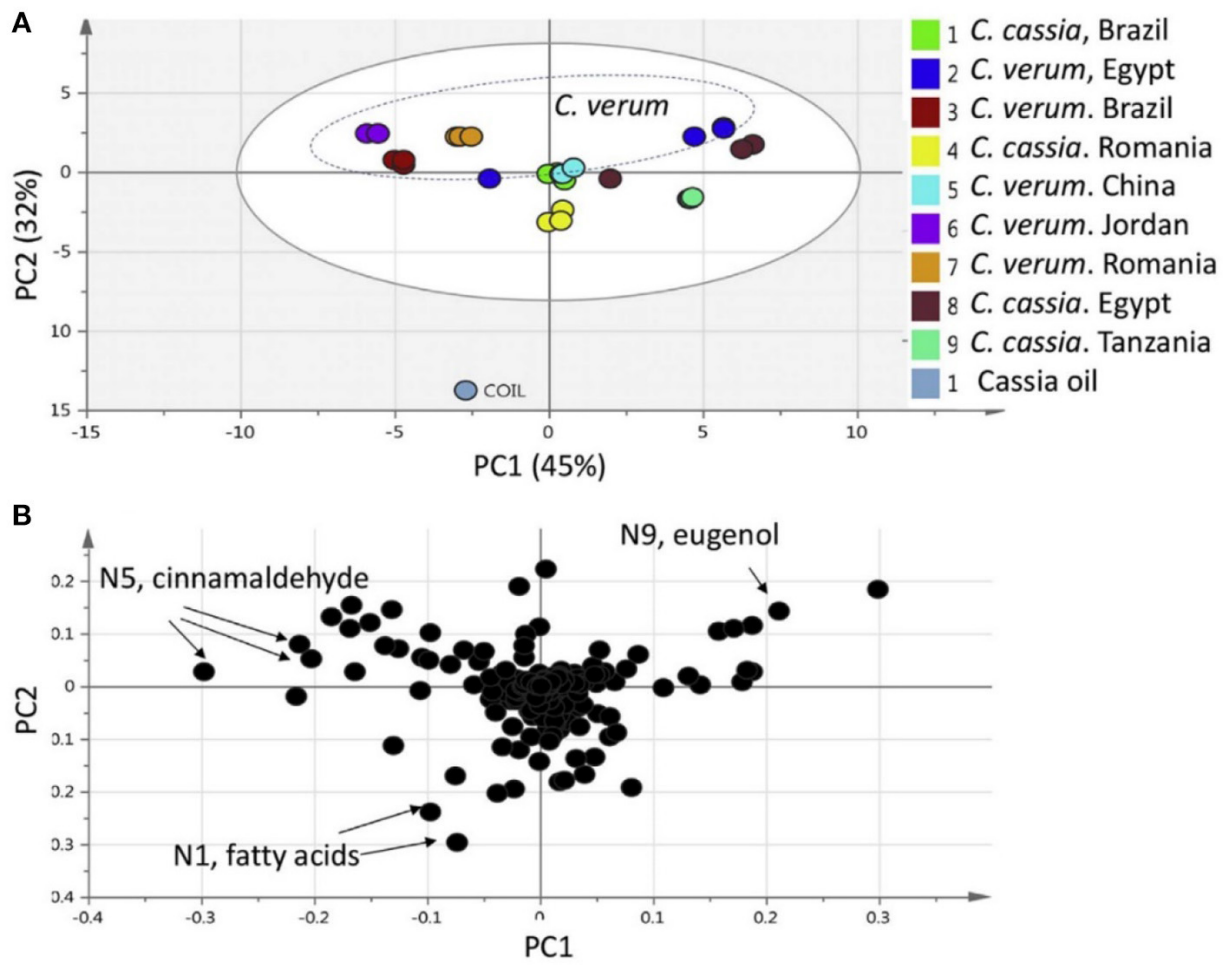
Principal component analysis (PCA) from Cinnamomum verum and C. cassia from different geographical origins, and representative commercial oil, using ^1^H-NMR (*n* = 3) metabolomics. The scores plot **(A)** demonstrates clusters at distinct spatial points in the PC1-PC2 scores plot, and loadings plot **(B)** highlights major contributing molecules to the separation of the samples. Reproduced with permission from Farag et al. (77). Copyright 2018, Elsevier Ltd.

Principal component analysis (PCA) is an unsupervised approach which projects multivariate data (with *k* features/variables) onto a smaller dimensional space (< *k*-1). As such, PCA is often referred to as a projection or dimension reduction method. The metabolite profile is reduced to uncorrelated principal components (PCs) which represent the total variation present in the metabolome. The first principal component accounts for the maximum percentage of the overall variance, the second principal component (orthogonal to the first) accounts for the second largest amount of variance, and so on until all the variation in the data is accounted for or the number of principal components reaches the limit (i.e., the number of features-1) (75). The principal components are plotted in a pair-wise fashion (typically the first two, which explain the most variation) on a 2-dimensional plane – known as a “scores” plot – that demonstrates the spatial relationship between different samples. Points which cluster together have similar correlations in the PC variations, which translates to similarities in their overall chemical profile. Likewise, dissimilar samples are located further from one another in the two-dimensional graph. A second corresponding graph associated with PCA is the loadings plot, in which the features (variables) are arranged in a two-dimensional plot using the same PCs as the scores plot. The spatial representation of the loadings mirrors that of the scores, thus enabling the determination of which features are more prevalent in certain clusters of samples. Zhang et al. (76) developed an approach to authenticate juices from different berry fruits using untargeted metabolomics. Using PCA generated from LC-QTOF-MS spectra, they were able to discriminate between blueberry, cranberry, apple, and grape juice (Figure 2). The corresponding loadings plot yielded 18 characteristic markers that were able to categorize the juices (76). Additionally, Farag et al. differentiated ten cinnamon accessions from the main cinnamon species using ^1^H-NMR metabolomics combined with unsupervised chemometric approaches (77). The scores plot (Figure 3) distinguished between *Cinnamomum cassia* and *C. verum*, with PC1 and PC2 comprising 77% of the variability in the model. The loadings plot suggested nine key metabolites which could be used to differentiate between cinnamon accessions, including cinnamaldehyde and eugenol; the exclusive presence of eugenol in *C. verum* samples suggested its potential as an authentication marker (77). Thus, PCA represents a robust and potent chemometric tool in the evaluation of different samples and their authenticity/purity.

However, while PCA can demonstrate clusters of samples based upon their chemical profile, it is not able to provide quantitative metrics around the degree of similarity between samples, nor ranking how similar samples are to one another. Furthermore, PCA relies on a subsection of the overall principal component model to visually represent similarities and differences between the samples; this is often an *ad hoc* choice of PCA components which can mask outliers or shift the overall spatial relationship between samples, leading to the possibility of specious results and subsequent conclusions. Integration of multiple PCs into a single quantitative comparison may circumvent this. Termed the composite score, it has potential to facilitate comparisons between multiple samples using the entirety (or at minimum a significant subset) of the principal component model to quantify similarity between samples (78). This approach was used recently by Wallace et al. to differentiate *Hydrastis canadensis* supplements from potential adulterants (79).

*Suggestions for future use:* PCA is a powerful unsupervised clustering tool with accessible computational resources to simplify analysis, making it an ideal first step in any chemometric analysis. PCA can be used prior to any supervised approach to confirm expected clustering among samples, and that apparent distinctions result from true variations in sample metabolomes, not as a result of overfitting to predefined categories. Alone, PCA can be used to determine if adulterated and pure samples differ while simultaneously identifying biomarkers likely responsible for any variation. Thus, PCA has potential for a quick and easy approach to botanical authentication based on metabolite profiles. Possible sample clustering that may be identified using PCA is species proximity, cultivation procedures, or origin of plant growth. For any authentication study requiring more detailed information of how samples are related or identification of unknown with a single model, other approaches should be performed concurrently with PCA.

#### Hierarchical Cluster Analysis (HCA)

**Figure 4:**
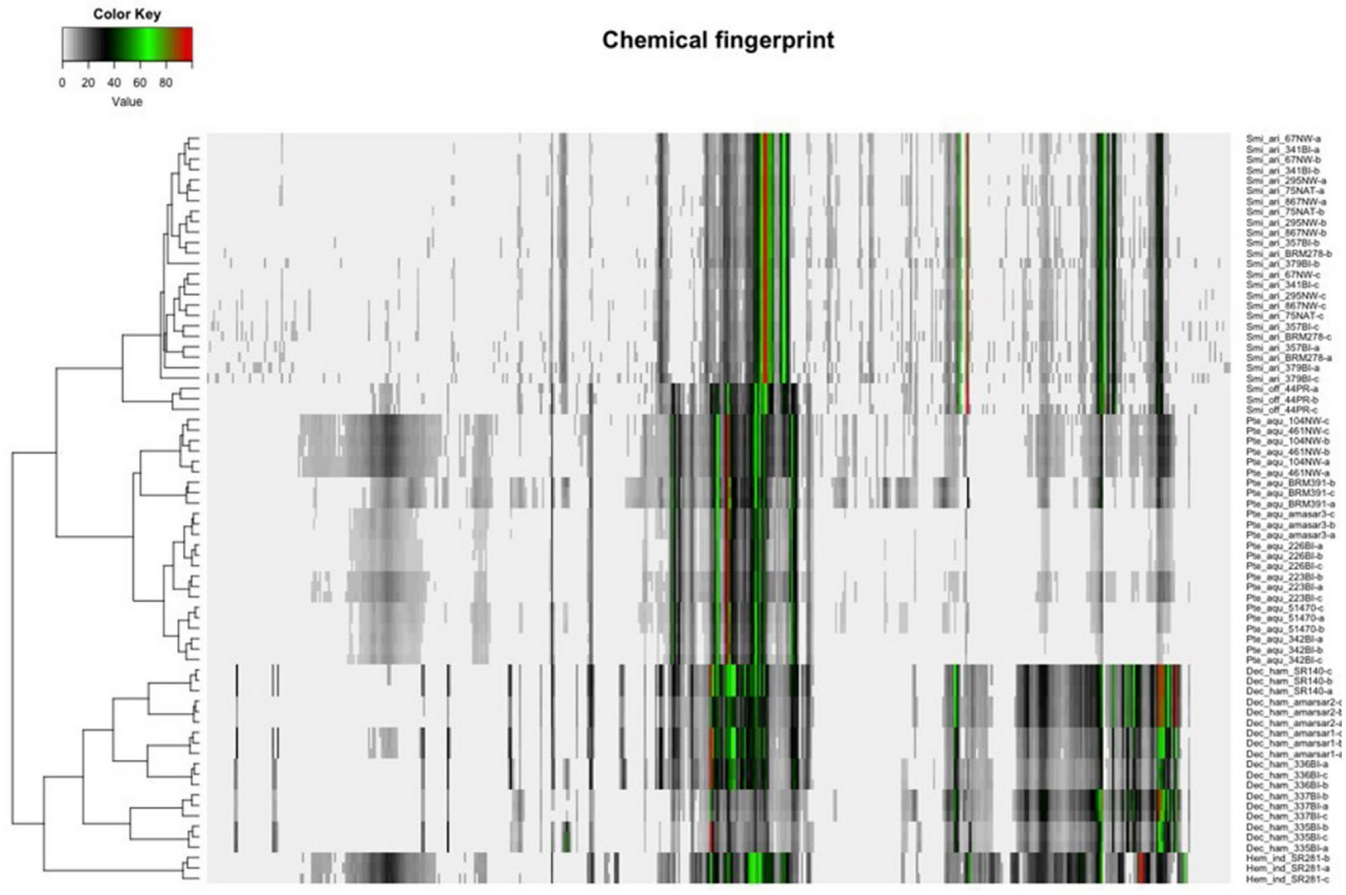
Unsupervised clustering analysis of four species of Sarsaarilla using cheometric modeling of ^1^H NMR data. Peak patterns are also provided to illustrate differences between four major species. Reproduced under a Creative Commons Attribution 4.0 license from Kesanakurti et al. (82).

Hierarchical clustering analysis (HCA) uses distances between sample groupings (clusters) to organize samples into taxonomies; objects with the highest similarity cluster together, and generated clusters are treated as a new, independent feature which are clustered with the next most similar variable. Similarity is calculated as distance between variables through a variety of algorithms, including Euclidian, Mahalanobis, or city block (Manhattan); similarly, there are various linkage rules for amalgamating the cluster analysis, such as minimum or maximum similarity between variables, group average (average similarity between every possible pair of data points), or Ward's Method (sum of the squared distance between each pair of data points). Proximity matrixes are used to compare the calculated similarity of all groups. The shorter the distance, the more similar the variable, and thus more likely to be related. However, since the similarity (distance) and linkage can be calculated using different combinations of rules, the results of cluster analysis are difficult to compare between studies. In the case of sample authentication, each botanical sample is treated as a variable and clusters are formed based on similarity in peak heights (or other metabolite features) so that the most chemically similar samples group together. HCA has been used to distinguish *Cirrhosae bulbus* from common adulterants using UPLC-ELSDA fingerprinting (80). Zhou et al. demonstrated the use of HCA in discriminating between two bitter melon (*Momordica charantia*) chemotypes with different medicinal properties (81). While PCA was able to distinguish the two chemotypes, HCA allowed a deeper insight into how each variety differed within the groups (81), and the combination of PCA and HCA predicted biomarkers for easy chemotype distinction of unknown samples. NMR chemical fingerprinting of Sarsaparilla species (*Decalepis hamiltonii, Hemidesmus indicus, Pteridium aquilinum*, and *Smilax* spp.) revealed four clear clusters, which were further confirmed by patterns in the NMR spectra (Figure 4) (82). In addition to detection of herbal adulteration, HCA provides opportunity to detect contamination with pharmaceutical drugs; Cebi et al. used HCA to classify coffee and tea blends adulterated with sibutramine, an illegal weight-loss drug (83).

*Suggestions for future use*: HCA models share similar possible directions as PCAs, with additional information of how samples are related chemically within generated clusters. A potential study may evaluate differences in chemotypic and genotypic based hierarchical clustering for authentication. It is possible genetic approaches may discover adulteration by non-target species but miss contamination with synthetic compounds; chemotypic approaches may simultaneously provide species distinction and chemical authentication. Similarly, comparisons of HCAs generated via multiple analytical techniques (H-NNR vs. LC-MS) may provide a deeper understanding of sample relationships though inclusion of additional compounds.

#### Self-Organizing Maps

Artificial neural networks (ANN) is a collective term for several machine learning methods. The most common unsupervised ANN approach is self-organizing maps (SOM). Section 5.2.3.4 provides an overview of supervised ANN in natural product authentication.

Self-organizing maps (SOMs), sometimes referred to as Kohonen maps or Kohonen networks, is a neural network-based algorithm that reduces the input dimensionality to represent sample patterns; SOM forms a 2-dimensional map where similar samples are mapped closer together. The benefit of this approach is that SOMs account for non-linear information in the data, and each variable's importance to the model can be derived from the weights associated with each map “point” (84). Torrecilla et al. (85) employed SOM to analyze extra virgin olive oils and detect adulteration via the addition of other oils. Using random and non-random noise to simulate adulteration, the SOM was constructed which yielded a misclassification rate <1.3% (68). Using previous research, Menezes et al. generated a library of terpenes present in three tribes of Annonaceae species (521 molecules) for use in training a SOM (86). The model was able to classify unknown samples into the three predefined tribes with 80% average accuracy (86). Similar approaches have been demonstrated using diterpenes to classify Lamiaceae spp. (87) and flavonoids to classify Asteraceae spp. (88).

*Suggestions for future use*: Menezes et al. provide an SOM method very applicable to natural product authentication (86). Using previous metabolomics data to classify botanical samples, despite variations in analytical and collection techniques, provides an opportunity to create authentication models without extensive benchwork. This approach should be applied to commercial supplements with well-defined chemistry to develop predictive models for existing products.

### Supervised Approaches

Supervised statistical methods require the data matrix have both independent and dependent variables, the latter of which can be nominal (categorical) or numerical in nature. Nominal dependent data are ideal for clustering data into pre-defined classes, such as “pure” and “adulterated,” whereas numerical data can allow for the ranking, quantifying, and comparing variables against each another. Many machine learning approaches are supervised models based on training datasets. Simply, a set of samples with known dependent variables are used to train, generate, and validate a model, which subsequently predicts the classification of additional, unknown samples (or the remainder of the data). However, as the numbers of samples in a metabolomics data set are generally fewer than the number of variables, supervised techniques are prone to overfitting the data (89); even so far as to be able to fit a model to completely random data (90). Therefore, model validation is critical before any interpretation of the model is reliable, and often quality criteria of the model are reported such as R^2^ (a measure of the fit of the model) and Q^2^ (the ability of the model to predict unknown samples) (91).

### Partial Least Squares (PLS)

PLS is a dimension reduction tool similar to PCA. PLS condenses complex data to simpler latent variables which explain shared features between correlated samples, but with a dependent variable to supervise the construction of the model. The goals of PLS are akin to linear regression: classification of dependent variables and understanding the independent variables (metabolite features) that are predictors of this classification. A PLS model plots the latent components among the independent variables that best explain variations in dependent variables, and samples are projected onto the model space. The resulting scores plot allows simple visualization of sample clustering based on the reduced variables; the loading plot provides information about specific variables which contribute the most covariance to the model. The two primary types of PLS analyses- PLS-R and PLS-DA are defined by the nature of the dependent variable.

#### PLS-R

Partial least squares-regressions model variations among the independent variables to explain a numerical dependent variable. While PLS-R is uncommon for quality control of botanical products, it has been employed with biomarker identification, biochemometrics, and detection of adulteration (92). For example, PLS-R was employed to differentiate between *Hydrastis canadensis* (goldenseal) and four common adulterants using FT-NIR data (64). Following preprocessing and filtering the spectral data, PLS-R modeling successfully clustered pure goldenseal from non-target species, as well as differentiated between various goldenseal parts (roots and shoots) (64). In this case, the plot consisted of latent variables which reduced the spectral data as guided by a gradient in contamination as the dependent variable. This study also highlights the importance of preprocessing and filtering data; unprocessed data was unable to distinguish species using PLS-R (80). Partial least squares is also one of the primary predictive chemometric approaches: when there are correlations are drawn between the dependent data set (often bioactivity or other quantitative data) and the independent chemical data from which the model is derived. This approach, known as biochemometrics when using bioactivity data, is explained more fully below.

*Suggestions for future use*: Some future applications of PLS-R in herbal product authentication could include evaluating products with known and specified variations in ingredients- teas with varying percentages of *Ilex paraguariensis* (yerba mate) and ashwagandha root for example. PLS-R may also be useful for discovering biomarkers to quickly differentiate between bioactive and inactive products though detailed bio-chemical analysis of commercial supplements and subsequently screening additional products for identified markers. This may bypass some typical issues with single marker analysis, as described in section Single Biomarker Approach, by using commercially available products for biomarker discovery as opposed to predetermined pure plants.

#### PLS-DA

**Figure 5:**
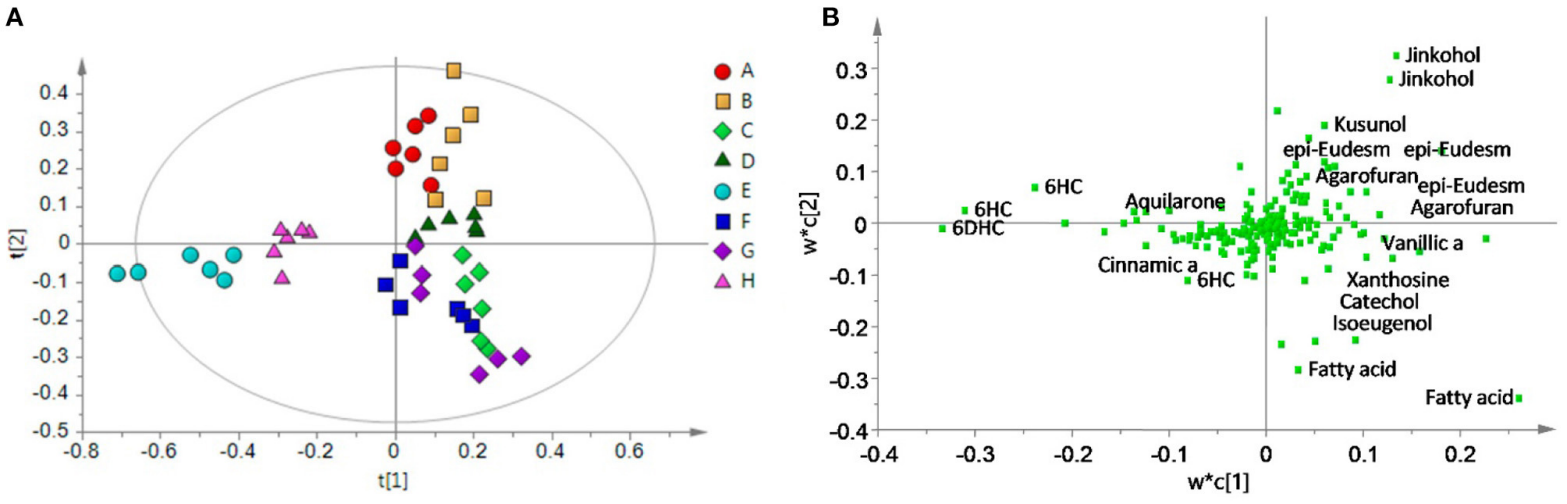
PLS-DA analysis of gaharu (Aquilaria malaccensis) woods by ^1^H-NMR untargeted analysis. The PLS-DA scores plot **(A)** effectively discriminated between lower grade products (“E” and “H”) and higher grades. And the corresponding loadings plot **(B)** demonstrated that the lower quality products contained higher levels of aquilarone derivates. Reproduced under a Creative Commons CC BY 4.0 license from Ismail et al. (93).

Partial least squares-discriminate analysis (PLS-DA) models the data similar to PLS-R, but with the caveat that the dependent variable be a binary descriptor (e.g., “class1” vs. “class2”, “authentic” vs. “adulterated”, etc.), which are coded as −1 and 1, or 0 and 1. The resulting scores plot is typically able to discriminate between the two groups, as it is guided by the classification of the samples. PLS-DA is one of the most common chemometric tools applied to chemical data for authentication and discrimination among botanical products. The study by Ismail et al. demonstrates this approach by differentiating between different grades of gaharu (agarwood, *Aquilaria malaccensis*). Using ^1^H-NMR metabolomics, a PLS-DA model was able to differentiate between “high grade” and lower grades of gaharu (Figure 5), and the resulting loadings plot also highlighted aquilarone derivatives that discriminated the different quality classes (93). Windarsih et al. also employed PLS-DA analysis to differentiate between authentic *Cucuma xanthorrhiza* (“Java ginger”) and samples adulterated with *C. aeruginosa*. PLS-DA yielded a robust model (R^2^ and Q^2^ of 0.993 and 0.986, respectively) which separated authentic from adulterated samples (94).

**Figure 6:**
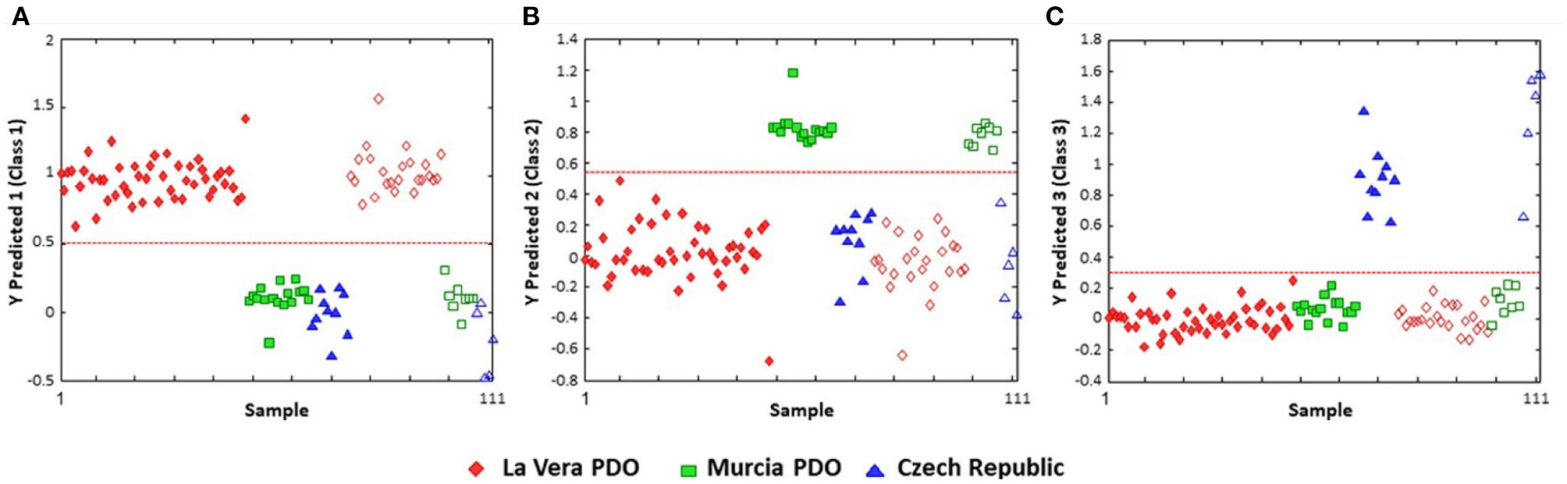
PLS-DA discrimination plots according to the geographical region of production of paprika. **(A)** La Vera PDO vs. other regions; **(B)** Murcia vs. the other two; **(C)** Czech Republic vs. other classifications. The dashed red line indicates the classification boundary between the two designations. Open symbols represent the training data; the solid symbols are the test data. Reproduced with permission from Barbosa et al. (95). Copyright 2020, American Chemical Society.

One of the limitations of PLS-DA is that the categorization is restricted to a binary class designation. If there are more than two main categories, the discriminant analysis requires pair-wise comparison, complicating the analysis and potentially limiting the conclusions which can be drawn. This is exemplified by Barbosa et al.'s study to differentiate and authenticate paprika grown in three different areas (La Vera and Murcia in Spain and the Czech Republic) (95). The PLS-DA classification plots were done as iterations of one region vs. the other two, to comprehensively demonstrate that the three regions were distinct from one another (a classification rate of 100%) (Figure 6).

*Suggestions for future use*: PLS-DA has excellent potential in herbal product quality control since binary categorical classes can encompass multiple facets of plant differentiation. These applications range from classifying samples based on geographic origin, plant parts, species or subspecies, or adulteration status. Although PLS-DA requires pre-determined classifications of data, the loading plots can guide discovery of biomarkers for quick screening of unclassified samples. An interesting study would model one chemical dataset for multiple classifications of the same samples to evaluate how clustering and model validation (R^2^ and Q^2^) change to determine the most reliable classifications for authentication.

#### Soft Independent Modeling of Class Analogies (SIMCA)

**Figure 7:**
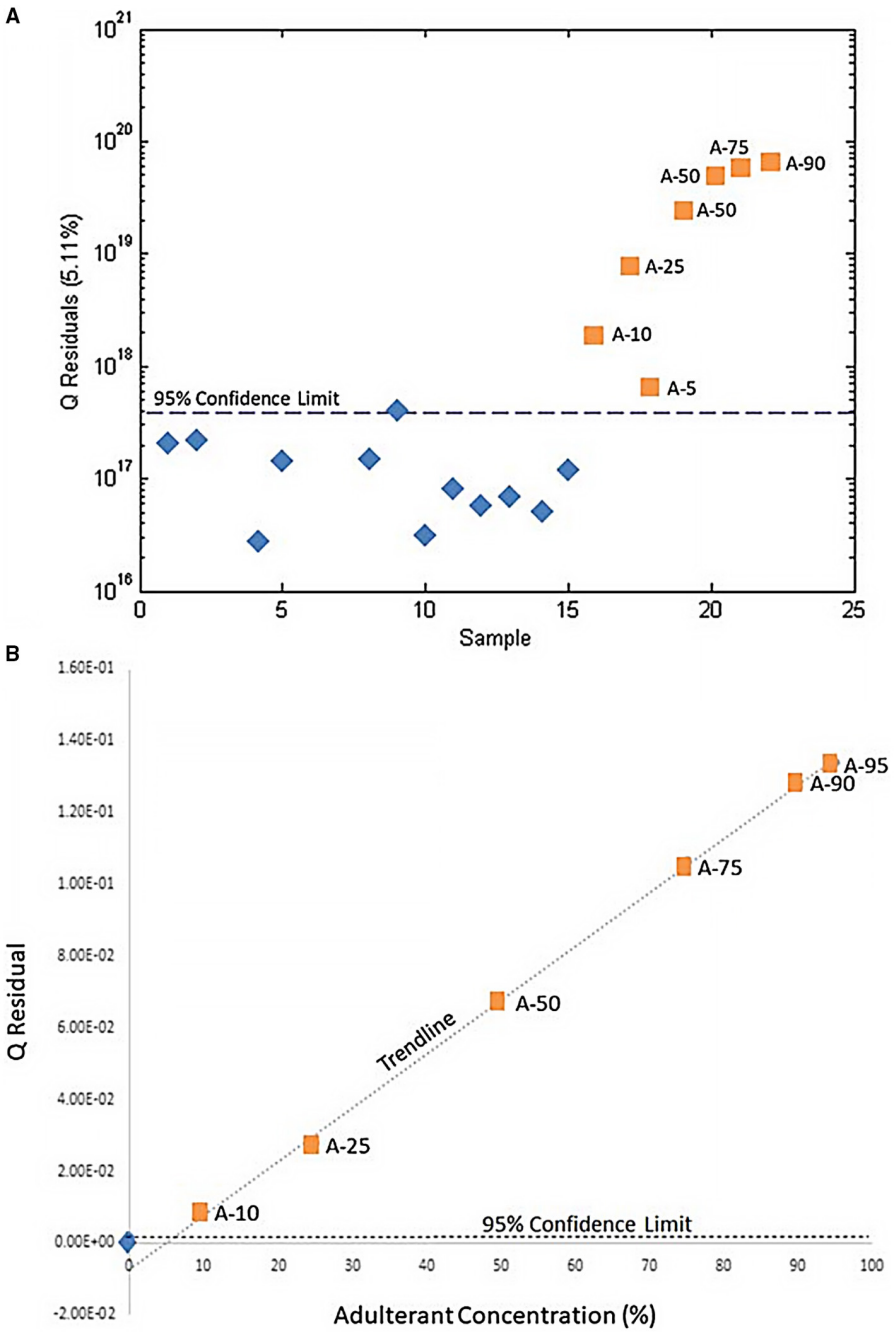
Use of SIMCA to determine adulteration of H. canadenis by C. chinesis. **(A)** SIMCA demonstrating that pure H. candenis samples (blue diamonds) are below the 95% confidence interval and adulterated samples (orange squares) are above the 95% confidence interval. **(B)** The Q-residual of each adulterated sample. The blue diamond represents the mean Q-residual for the unadulterated H. canadenis samples. Reproduced with permission from Wallace et al. (79). Copyright 2020, Springer Nature.

SIMCA is a supervised expansion of PCA: samples are grouped into predefined classes and PCA is performed on each class, so that each group is projected onto a separate PC space. To detect adulteration, there are only two classes: authentic or adulterated, so one-class PCA's for authentic or adulterated samples can be generated (79). A new, unknown sample's classification is predicted by projecting it to the PC space and calculating the Q statistic (or Q residual), quantifying the similarity of the unknown PCA to the training set's PCA (79). The Q-statistic predicts if the new sample belongs in the authentic, adulterated, both, or neither class. Thus, SIMCA distinguishes similarities among samples and unknowns rather than defining the differences between groups (96). Wallace et al. intentionally adulterated *Hydrastis canadenis* with varying concentrations of *Copits chinesis* and used untargeted metabolomics with SIMCA analysis to differentiate between pure and tainted samples. Using one-class modeling, the Q statistic of “unknown” adulterated products was calculated and found to fall above the 95% confidence interval for pure samples, successfully identifying even the lowest percentage of contamination (5%) and providing a higher resolution of differentiation than PCA alone (Figure 7) (79).

*Suggestions for future use:* SIMCA is a powerful classification tool with digestible graphic outputs. SIMCA can be used for classification problems where the output for each sample is already known, such as adulterated vs. pure. It is a straightforward tool for analysis of binary classifications but becomes more complicated as more categories are added. Thus, it should be reserved for problems focused on identifying contaminated samples when deep machine learning modeling is not necessary. A suggested approach to botanical quality control is to perform unsupervised PCA to identify and confirm a binary clustering of samples followed by SIMCA to predict the classification of unknown products.

#### Machine Learning Models

While easily interpretable, models such as SIMCA and PLS are inherently linear algorithms, capable of modeling only linear latent covariance. As biological data are often non-linear, it is probable that the related chemical data also has a non-linear latent structure. Thus, non-linear machine learning methods can be uniquely suited to examine relationships from metabolomic or other chemical data.

#### Decision Trees

**Figure 8:**
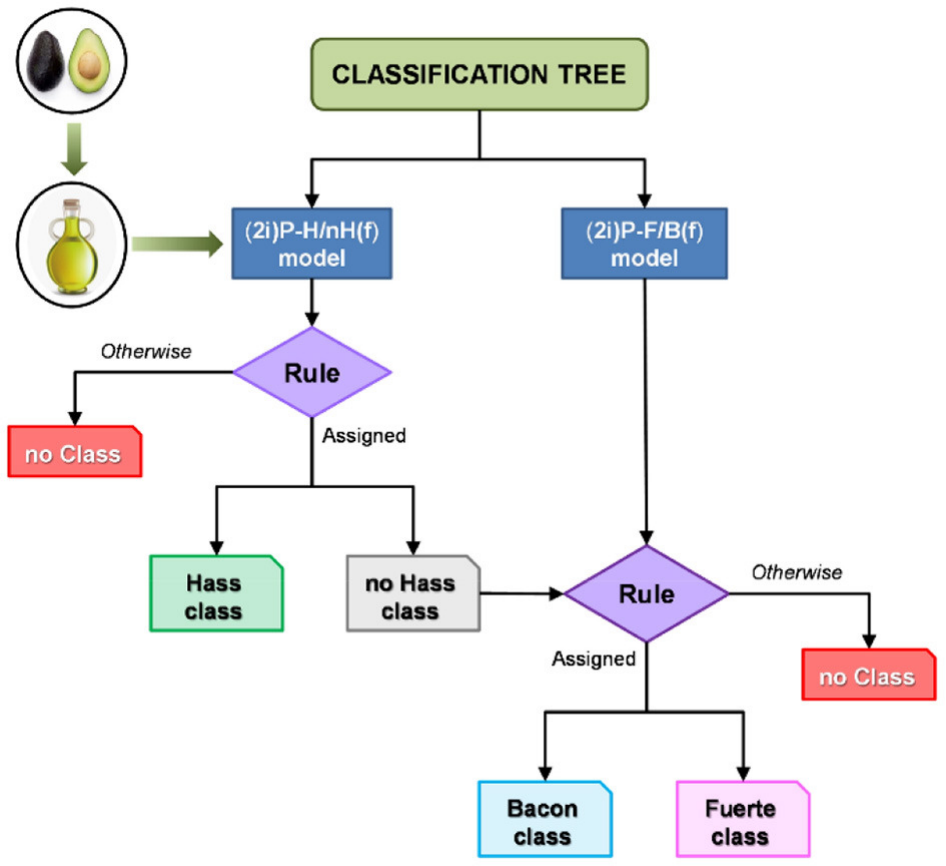
Use of classification trees to identify unknown avocado oil samples as a specific cultivar or as no class based on HPLC-CAD metabolite profiles of a training set. Reproduced with permission from Martin-Torres et al. (97). Copyright 2019, John Wiley and Sons.

Decision trees are a machine learning approach that use hierarchical decisions to determine sample classification based on training data. Trees are displayed upside down, with the bulk data at the top being split based on features that best distinguish the data at each step. These distinctions are typically based on the presence or ratios of specific metabolites that separate one classifier from another. The result is a tree split into branches at decision nodes that end with leaves, or the classification groups. Decision trees are commonly referred to as classification and regression trees (CARTs) to encompass both distinct variables (classification) and numerical or continuous variables (regression). In the case of botanical product quality control, samples are classified based on species, purity, or other relevant factors. Classification trees were used to classify different cultivars of avocados based on HPLC-CAD metabolomics (97). Training data that comprised of spectra from 32 avocado samples of three varieties generated a tree which guided classification of unknown avocado oil samples into cultivar classes or no class based on specific, model generated rules (Figure 8) (97). A strength of decision trees is the ability to classify an unknown sample into a “no class” group to avoid overfitting or forcing a sample into a classification group. A creative application of decision trees is to predict the need of specific safety tests and evaluations for botanical products, as demonstrated by Little et al. the group used an *in silico* decision tree model to analyze the need for safety assessments of botanical products based on UHPLC with UV, CAD, and HRMS metabolomics, structure identification, consumer exposure, and existing safety evaluations (98). The developed tree used chemical data and previous records to determine if any tests are necessary for consumer safety depending on the presence of certain metabolites in the sample and database information of safety data (98). This study highlights the versatility of decision trees in quality control – they can not only identify botanical adulteration, but they can also ensure safe practices while developing botanical supplements.

*Suggestions for future use:* Decision trees have the appeal of a visually appealing output for a complex machine learning model. They hold promise for discerning unknown product identification, detecting adulteration of products with known contaminants, and discovering biomarkers for various classifications. Once the decision tree model is built with training data, it is relatively straightforward to feed an unknown's chemical data through the model to predict its classification. This is an exciting possibility for quality control, especially when the most common adulterants are known to base relevant decision trees around. It should be noted that the decision algorithms at each node are based on separating the data available from the previous split, but the split progressions may not actually be the most reliable representations of divisions in the data. Random forests (described below) increase the accuracy of node splits but lose clear visual representation of the model.

#### Random Forests

Developed in 2001 by Breiman (99), random forest (RF) methods build an ensemble of decision trees, each of which is trained using the dependent variable(s). Each tree produces an outcome, and the aggregate outcome from all the trees (aka the forest) is reported as the outcome of the model. Random forest holds several advantages over other methods. The multiple decision trees produce more accurate classifications compared to a single decision tree algorithm, and it is less prone to overfitting than other supervised approaches (100). Additionally, one other advantage of random forest is that it can be used for both classification and regression problems. Deklerck et al. used random forests to classify heartwood samples of *Pericopsis elata*, a protected timber species. Using Direct Analysis in Real Time ionization coupled to time-of-flight mass spectrometry (DART-TofMS) on wood slivers, the random forest model using cross-validation was able to correctly predict *P. elata* samples (101). To analyze *Zanthoxylum* seed oils, Houet al. built a random forest classification model that differentiated between the two main species (*Z. bungeanum* and *Z. armatum*) with 100% accuracy from cross-validation. Even simplifying the model to only the most important chemical features, the cross-validated model still maintained 100% accuracy (102). Random forests also have the ability to be a predictive machine learning tool, and provide correlative predictions between dependent variables and the associated independent chemical dataset (103).

*Suggestions for future use:* Random forests can be used for the same purposes as decision trees where increased reliability of decisions at each node is necessary. This includes instances of fewer chemical data or a smaller number of training samples. Since random forests combine multiple decision trees, the computational input is much higher, so model building and training takes longer. Thus, random forests are not the best option when expecting a quick turn-around. However, there is potential application in developing random forest models for detection of adulteration of complex botanical products and mixtures. The extent of random forests in detecting contamination and purity of extremely complex samples in a high-throughput manor should be explored.

#### Support Vector Machine

Support vector machines (SVMs) are another supervised machine learning technique that can be employed for regression or classification analyses. The objective of the SVM algorithm is to find a plane in a *k*-dimensional space (*k* representing the number of features) that distinctly classifies the data points into groupings so that it has the maximum margin (i.e., maximum distance) between data points of both classes. Similar to other supervised machine learning or multivariate approaches of chemometrics data (where the number of features outstrips the number of samples), SVM can be prone to overfitting, so training on a smaller subset of samples, followed by cross-validation, is key to generating robust classification or predictive models.

**Figure 9:**
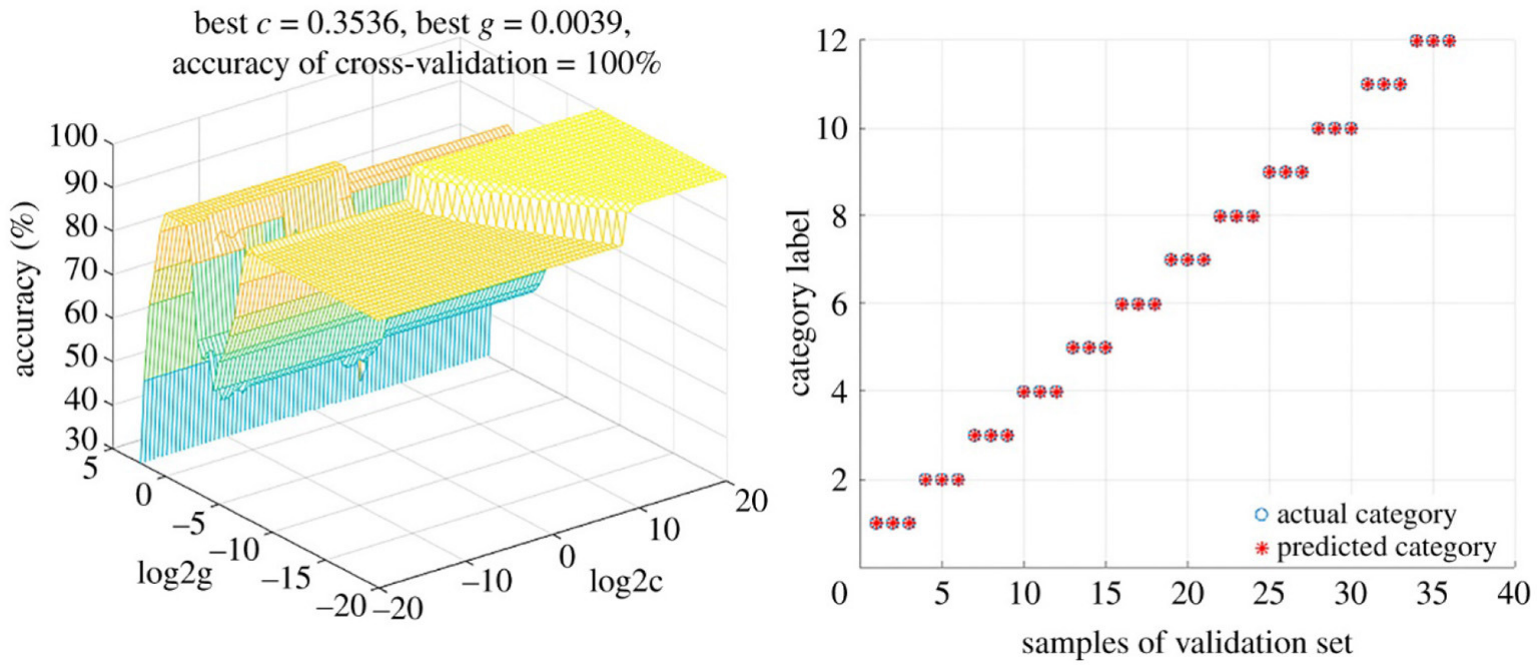
Support Vector Machine (SVM) model for differentiating between Dendrobrium species. Using a low-level fusion strategy of MIR and NIR spectral data from 12 Dendrobrium sp. Reproduced under a Creative Commons Attribution License 4.0 from Wang et al. (107).

Martín-Torres et al. (97) used SVMs to differentiate between the geographical origins as well as the botanical variety of avocados. Samples from five different countries (on three continents) representing seven avocado varieties were analyzed using normal phase HPLC-UV/VIS, after which the data was interpreted using two different multivariate approaches. The authors found that PLS-DA and HCA were unable to resolve the differences in geographical origin or between main groupings of variety. However, a three-input class SVM classification model (3iC) correlated the three different continents of origin (Africa, Americas, and Europe), as well as between the three dominant varieties (“Bacon”, “Fuerte”, and “Topa-Topa”); the latter having 100% correct assignments and precision and sensitivity of 1.00 (104). SVMs were also used to classify *Paris polyphylla* via fusion of Fourier-transformed infrared spectroscopy (FTIR) and UV-VIS spectroscopy data (105). Pan et al. used untargeted LC-MS metabolomics to profile five different *Uncaria* species in order to authenticate the source of Uncariae Rammulus Cum Uncis (Gou-Teng). A SVM model correctly categorized both training and test samples, and was used to classify 20 commercial Gou-Teng (GT) samples (106). The model predicted 16 of the samples were *Uncaria rhynchophylla*, while four did not match any of the *Uncaria* species. These four samples exhibited LC-MS chromatograms that were substantially different from the others, and thus it was believed that these were other *Uncaria* species or mixtures of *Uncaria* species. This represented a significant advantage over other (un)supervised techniques for discrimination purposes. And using data fusion techniques of mid-infrared (MIR) (transmission and reflection mode) and near-infrared (NIR) spectra followed by SVM analysis facilitated the discrimination of 12 different *Dendrobrium* species (107). SVM provided perfect discrimination (100% accuracy rates) for both calibration and validation sets (Figure 9).

*Suggestions for future use*: SVMs have practical applications for both classification of test data and prediction of unknown samples origin, species, or cultivar. SVMs may prove useful for distinction of genetically and chemically similar plants that cannot be differentiated by other clustering models, either supervised or unsupervised. For example, many sub-species of herbs have overlapping genotypes due to crossbreeding and PCA analysis can fail to separate the most closely related cultivars using chemical data (108); SVMs may provide a deeper level of distinction. This application can be applied to authentication of products commonly adulterated with very similar species that lack the promised medicinal output. Since SVM models can automatically handle missing data, SVMs can be used for metabolomes with variable metabolite profiles and lower resolution analytical techniques.

#### Genetic Algorithms

Genetic algorithms (GA) are based on the processes of evolution, including natural selection, reproduction, and mutations. These processes take place over multiple generations of increasingly accurate and simple solutions to a complex problem (109). In the case of botanical authentication and quality control, the problem may be product identification, detection of adulteration, or biomarker discovery. The solutions are the subset of metabolites and their ratios that best classify samples based on predefined classes or distinctions. As a brief example, consider Gil et al.'s study which used a GA to identify the region of rose wine origin (110). At generation 0, every combination of the 79 polyphenols present in the samples as detected by UPLC-MS were evaluated for their ability to distinguish between origin region. Solutions with the highest fitness, or its distinguishing power as determined by linear discriminant analysis, were selected for reproduction. During reproduction, two solutions were mixed in a cross-over like process to create a new generation of unique solutions with higher fitness than the previous. The selection and reproduction processes were repeated for five generations, and each of the final combinations of polyphenols was tested for its accuracy in cross-validation tests. Those with the highest accuracy were further evaluated for their ability to discriminate wine origin regions in an unknown validation sample set. The GA model was able to discover a set of 4 polyphenols that had 86.7% accuracy (110). GA also provides the opportunity for simultaneous sample and variable selection for improved speed and accuracy for unsupervised clustering of samples and biomarker discovery (111). This bi-clustering approach opens the door for high-throughput metabolomics authentication of botanical materials.

*Suggestions for future use*: Potential for GA in botanical product quality control ranges from geographic identification to generation of a subset of biomarkers for subsequent analysis. The speed of GA modeling is ideal for situations requiring fast turn-around, so it is practical for developing authentication schemes for new products or products with increasing rates of adulteration. It should be noted, however, that GAs can be difficult to interpret, since the steps the model takes to combine data and reach a solution are not defined for the user and models fed the same data often reach different solutions. Thus, users should only use GA when the intermediate steps are not necessary for model validation.

#### Artificial Neural Networks (ANN) With Known Outputs

**Figure 10:**
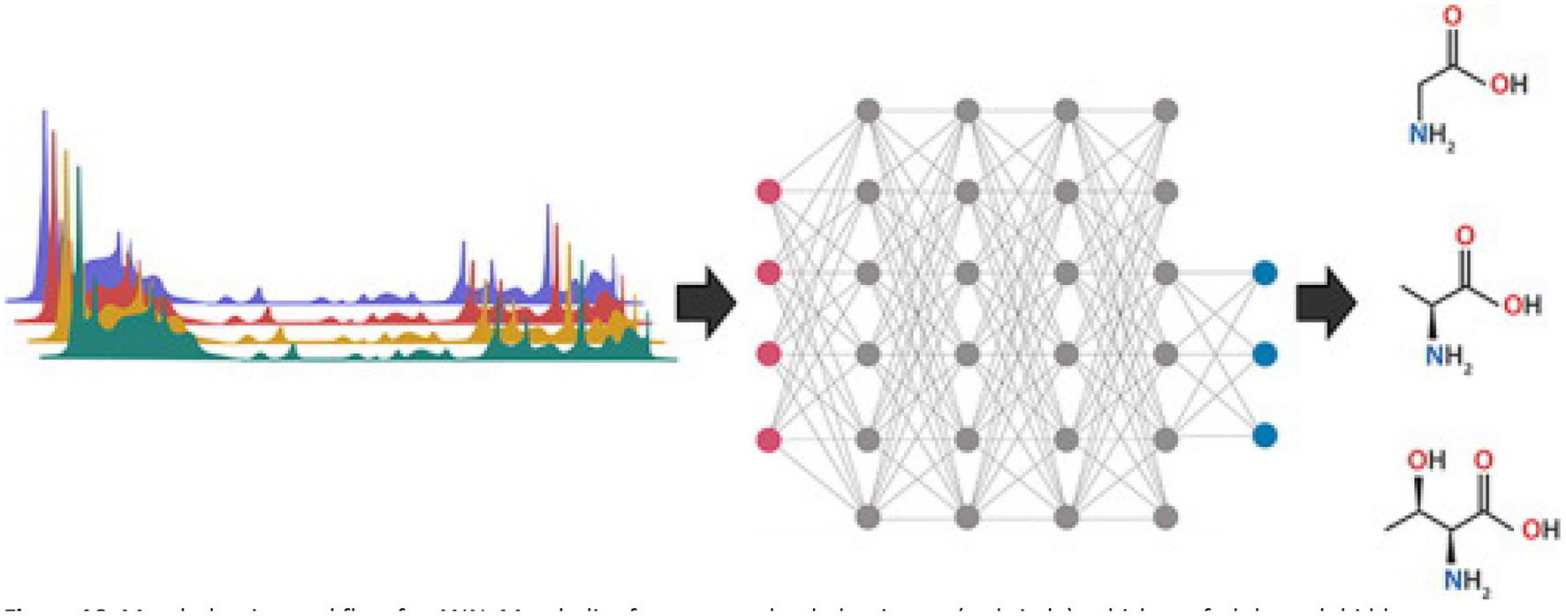
Metabolomics workflow for ANN. Metabolite features are loaded as inputs (red circle), which are fed through hidden neuron networks (gray circles), and categorized into output categories (blue circles). Each connection (gray line) has a weight, and each neuron has a bias, which are used for the activation functions. Reproduced with permission from Pomyen et al. (112) Copyright 2020, Elsevier Ltd.

ANN are the backbone of deep learning machine learning models. Mimicking the human brain and neurons allows computers to recognize complex patterns in sets of training data and predict the classification of a new dataset using the resulting model. There are three main sections of an ANN: an input layer, an output layer, and hidden layers in between (Figure 10) (112). Each metabolite from the complete set of samples is treated as an individual input and connections are generated randomly through multiple hidden layers to generate an output response. Hidden layers are comprised of “neurons” that connect metabolites with a random numeric weight and have a randomly assigned bias (Figure 10) (112). Together the weights and bias generate an activation function to determine if a neuron will be activated for use in the next hidden layer. This process of forward progression is repeated until the model predicts an output (such as adulterated or pure). Typically, the first prediction is incorrect since the weights and bias are random, so the model uses backwards progression using the prediction error to modulate weights and biases throughout the hidden layers. Through multiple rounds of forward and backward progression with a variety of inputs belonging to each output category, the model can predict the output of new data by processing the new inputs through the meticulously developed hidden layers (112, 113). The history of ANN in metabolomics, as well as an in-depth explanation of different ANN models for spectral data is reviewed by Mendez et al. (113).

Binetti et al. used ANN with merceological, NIR, and H-NMR data to classify olive oil cultivars (114). Using H-NMR spectral data, ANNs were able to classify unknown samples with >99% accuracy, despite variable environmental, harvesting, and processing conditions (114). Additionally, ANN modeling of headspace solid-phase microextraction (HS-SPME) coupled with GC-TOF-MS of 374 honey samples over two years provided 94.5% accuracy in prediction of honey origin when data from both collection years was combined (115). These studies are promising for herbal product classification - botanical material analysis is typically complicated by temporal, environmental, and procedural variations. In addition to classification and identification, ANN modeling has potential to predict the chemical and medicinal properties of supplements without extensive bioassays and robust chemical profiling (116). Using species classification and extraction procedures as inputs, Tusek et al. used an ANN to predict chemical features, including total phenolic content and extraction yield, and antioxidant potential of nine medicinal plants (116).

*Suggestions for future use:* ANNs hold immense potential for herbal product authentication. Since training data covers a range of environmental, temporal, and procedural variables, the predictive nature of the resulting model has very high accuracy. This is critical for commercial products that have limited information about harvest and processing procedures. An interesting study would determine if a single ANN model built on samples with a range of preparations (powdered, dried, capsules) and environmental factors can successfully classify and authenticate various types of new products. Additionally, prediction of medicinal properties using ANN should be expanded to allow confirmation of desired effects from commercial products quickly and accurately. This will take authentication a step further from identifying product constituents and increase efficacy of botanicals on the market. Users should take caution when using ANN to not over interpret their results. While ANNs are powerful classification tools for large data sets, they do not provide information on the chemical distinctions on which the model is built. Thus, the model does not allow interpretation about specific chemicals responsible for classification of samples.

### Precautions for Using Classification Models

Each model describe above has benefits to the natural product community, and there are examples highlighting their usefulness in the literature. However, each model also has pitfalls. It is crucial for researchers to understand the dangers of overinterpreting their outputs. One such downfall is overfitting data, or forcing data points into a category due to the lack of a “unknown” output option within the model. Almost all of the models described in this review are prone to overfitting, but some models, like decision trees and random forests, reduce this possibility by including an unknown option or compiling the output of multiple models into the output. It is important to validate each model by withholding a sample's data as a validation set with a known output, as well as reporting the Q^2^ and R^2^, as described in section Unsupervised Approaches.

An additional warning is that not every model is applicable in each situation. Despite a model seeming to fit a research goal, it is possible the type, quantity, or quality of data is not applicable to a given algorithm. Multivariate statistics and metabolomics projects require careful planning prior to data collection to ensure desired models can be used. For suggestion of models to use in different situations, see section Conclusions and Future Directions.

## Combining Orthogonal Datasets

While modeling chemical data through chemometric approaches can leverage the immense information contained therein to investigated nuanced differences between samples, being able to differentiate samples based on their geographic origin, taxonomic relationship, or adulteration level, the chemical composition represents only one facet of potential data to be analyzed. Incorporating additional data sources, whether it is from orthogonal chemical analyses, bioactivity/toxicity data, or genetic data, has the potential to develop discriminatory models that are even more robust in authenticating botanical products. Often, combinational approaches can increase the efficacy and reliability of natural product quality control, and should be implemented when feasible.

### Multiple Chemical Analyses Inputs

There is no single chemical analysis able to profile every metabolite present in a complex sample; each approach has some detractors. Ultra violet-visible spectroscopy (UV-VIS) requires a chromophore that can absorb energy within these wavelengths of light (often 180–800 nm); mass spectrometry (MS) can only monitor structures that are ionizable; nuclear magnetic resonance (NMR) is not as sensitive in detecting low-abundance compounds (55, 117). Therefore, combining different chemical investigations of a metabolome can better represent the chemical diversity present in a sample, and consequently allow for more precise modeling and differentiation between samples. These ‘data fusion' approaches have been used with different botanical products to evaluate their authenticity and detect adulteration. Spiteri et al. combined 1H-NMR with LC-MS to discriminate between commercial honey. The PCA was constructed considering each technique separately, and then combining NMR and LC-MS together. The authors found that the discriminating potential increased through data fusion, allowing better separation of the four different floral origins with no misclassification observed (118). NMR and LC-MS were also combined to detect adulteration of a commercial botanical dietary supplement which had resulted in the hypotensive collapse of several consumers. The product was purported to contain the species *Crataegus oxyacantha, Olea europea, Capsella bursa-pastoris*, and *Fumaria officinalis*. However, the analysis revealed the presence of indole alkaloids belonging to the genus *Rauwolfia*, such as ajmaline, reserpine and yohimbine. Subsequent quantitative analysis determined reserpine was present in pharmacologically-relevant doses (119).

Chemometric analyses using multiple analytical inputs have also been used to elevate and extract more information from more common and less expensive analyses, such as infrared analysis (IR) and ultraviolet-visible spectroscopy [UV-VIS, often abbreviated as LC methods (HPLC or UPLC) or diode array detectors (DAD)], to provide robust data and allow clear discriminate model formation. Combining three different types of detectors: diode-array detection, evaporative light scattering detection and mass spectrometry, Deconinck et al. constructed fingerprints for three common herbal products—*Rhamnus purshiana, Passiflora incarnata* L. and *Crataegus monogyna*. Using unsupervised projection chemometric analyses, the researchers were able to detect the presence of these plants in three different herbal matrices as well as in commercial preparations containing multiple botanicals (120). Wu et al. reported that fusing data obtained from polyphyllin content, FTIR spectra, and UPLC chromatograms yielded correct discrimination of Paridis Rhizome samples according to botanical and geographical origins by PLS-DA modeling. The authors reported that SVM and RF provided similar results (105). Two-step fingerprints, built upon mid infrared spectroscopy (MIR) and HPLC chromatograms, were analyzed by k-nearest neighbors and SIMCA to screen for five regulated plants used in commercial dietary supplements (121). And Zhou et al. fused two different infrared technologies – Fourier transform mid-infrared (FT-MIR) and near infrared (NIR) – to detect the origin of 210 *Panax notoginseng* samples from five cities in Yunnan Province, China. Random forest was used to establish classification models, which resulted in a classification accuracy of 95.6% (122). Data fusion of orthogonal analytical approaches has the potential to cover complementary facets of chemical space, and the subsequent modeling can be seen to be more powerful in its ability to discriminate between botanical samples as a means of authentication and adulterant detection.

### Biochemometrics

**Figure 11:**
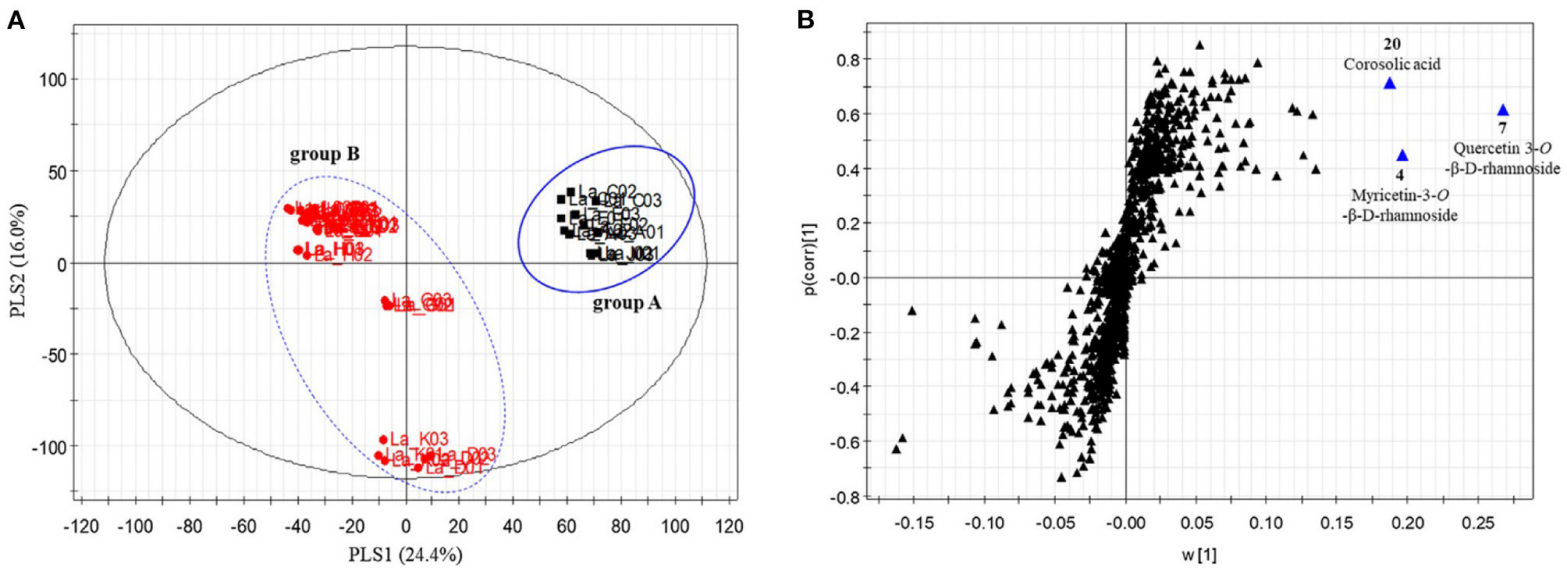
Partial least squares-discriminate analysis of Lagerstroemia samples. Scores plot **(A)** from the PLS-DA model accounted for 40.4% of model variability, and demonstrated two distinct clusters of samples. The S-plots **(B)** revealed two flavonol glycosides (myricetin-3-O-β-D-rhamnoside and quercetin 3-O-β-D-rhamnoside) and corosolic acid as potential discriminatory biomarkers with activity in stimulating glucose uptake. Reproduced with permission from Kim et al. (133). Copyright 2020, Elsevier Ltd.

The ability to profile large swaths of a metabolome without iterative methods of separation and purification means metabolomics approaches have an advantage for screening for bioactive metabolites (92, 123, 124). Integrating metabolomic fingerprinting with biological activity data allows for supervised methods to statistically model correlations between variations in biological response with differences in chemical composition across samples. These methods, collectively known as “biochemometrics”, have become a driver for bioactive molecule discovery. Several statistical methods have been utilized for this purpose, including hierarchical clustering analysis (125), partial least squares (92, 126, 127), and partial least squares-discriminate analysis (128, 129). Of these, PLS and PLS-DA have emerged as the foremost multivariate approaches for biochemometric analysis. These approaches utilize different variable metrics to ascribe correlation (and thus importance) to the chemical signals with the variable importance in projection (VIP) plot, the S-plot, and the selectivity ratio being among the leading metrics (92, 130–132). Biochemometrics holds great promise for botanical examination and authentication, as it could leverage relevant biological activity to determine a targeted fingerprinting method which has relevance to the biological function of the plant (as opposed to *ad hoc* choices of metabolites). Kim et al. used a biochemometric method to evaluate 17 different species of grape myrtle (*Lagerstroemia* spp.) based on their ability to increase glucose uptake *in vitro*. From the PLS-DA model (using the glucose update as the dependent variable), the *Lagerstroemia* sp. were grouped into two clusters, and from the S-plot the authors identified three main metabolites (myricetin-3-O-β-D-rhamnoside, quercetin 3-O-β-D-rhamnoside, and corosolic acid) that predicted glucose uptake activity and could be used as a discriminatory model for identifying bioactive species from the genus (Figure 11) (133). The integration of biological activity as an orthogonal dataset, and as a continuous numeric dependent variable in the dataset, allows for supervised chemometric methods to provide greater interpretation of the discriminatory model creation and identification of bioactive components. This can lead to the development of fingerprinting or authentication tools that correlate with the relevant biological effects of the botanical in question.

### Multi-Omics Integration for Botanical Control

**Figure 12:**
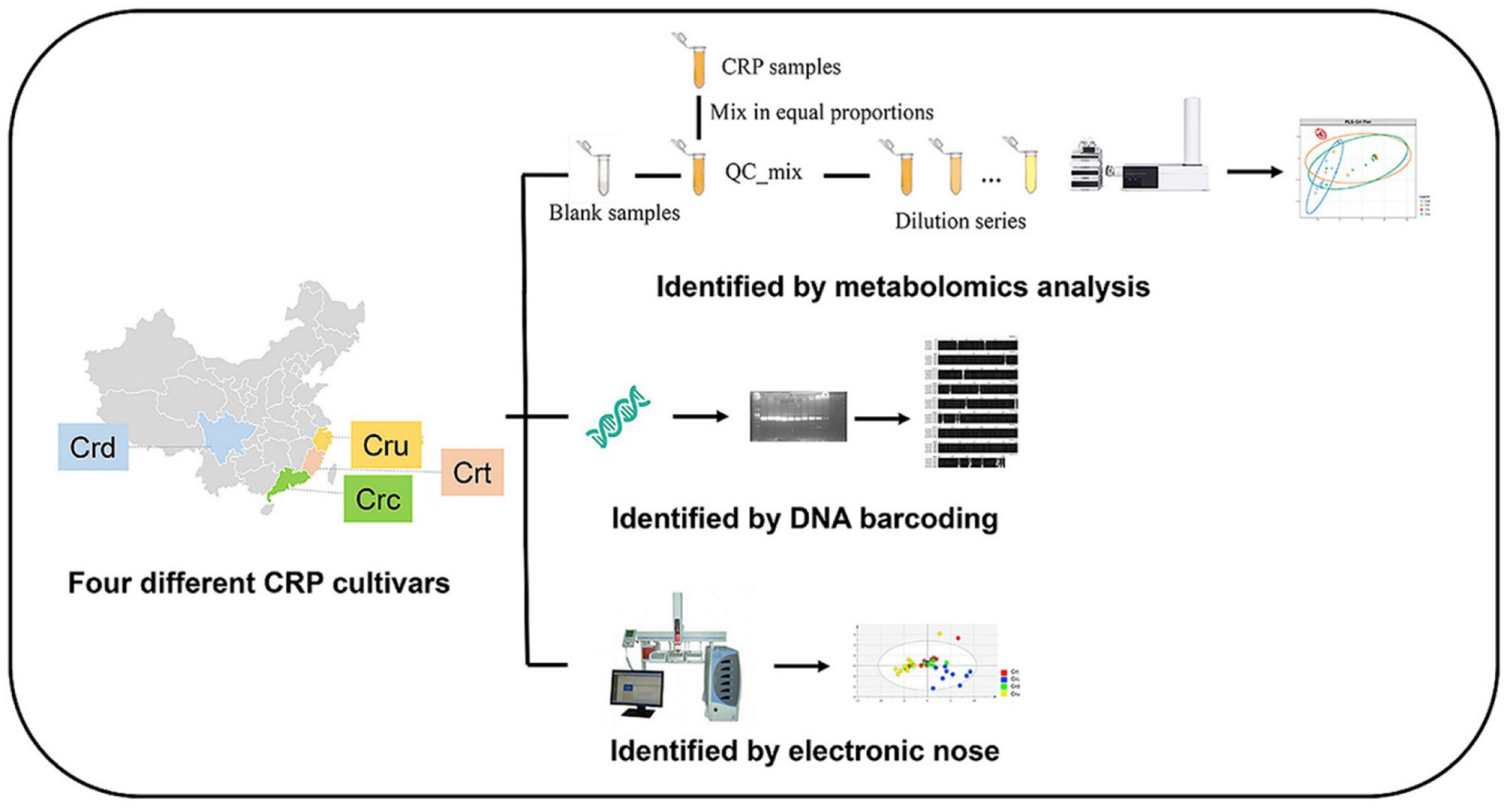
Integration of metabolomics, DNA barcoding, and electronic noise increases accuracy of Citri Reticulatae Pericarpium cultivar distinction compared to each method alone. Reproduced with permission from Li et al. (135) Copyright 2020, Springer Nature.

While metabolomic approaches provide ample opportunity for accurate, robust, and time-efficient authentication of complex botanical products, combining chemical data with other -omics approaches may yield the most effective solutions. Most commonly, metabolomics modeling is combined with genomics data. As mentioned in section Genetics, DNA barcoding and metabarcoding can lose accuracy at the species and subspecies level. Similarly, clustering of metabolites can lose resolution of chemically similar plants. Combining DNA barcoding using *rpoC1* and LC-MS metabolomic fingerprinting allowed species-level distinction between nine Phyllanthus species (134). Integration of genetics and metabolomics is easily the most common approach to botanical product identification, as outlined in Table 1. There are instances when metabolomics and genetics in combination cannot differentiate between species, so additional analytic approaches are employed, such as electronic noise (141), microscopy (142, 143), high-resolution melting analysis (144), Raman spectroscopy (145), or multiple metabolomic approaches (146). Figure 12 demonstrates that integrating multifaceted -omics approaches can be achieved in a single study to increase the power of distinction and authentication of herbal products (141).

**Table 1:** Orthogonal approaches to integrate genomics and metabolomics data analysis of botanicals.

**Botanicals**	**Product type**	**Genetic approach**	**Metabolomic approach**	**Modeling**	**Author**	**Year (Ref.)**
**Qin jiao**	Dried root powder	ITS2 barcoding	Q-TOF-MS	ANOVA	Li et al.	2020 (135)
			H-NMR	PCA		
				OPLS-DA		
***Hypericum*** **taxa**	Essential oil	ITS2 barcoding	GC-MS	PCA	Zeliou et al.	2020 (136)
	Dried leaf	ITS1 barcoding	LC-HRMS	Biplots		
			LC-DAD-MS	Mantel test	
			HPLC-DAD			
***Salvia*** **subg** ***Perovskia***	fresh leaf and root	trnH-psbA barcoding	UHPLC-QTOF-MS	PCA	Bielecka et al.	2021 (137)
		ITS2 barcoding				
***Hypericum*** **spp**.	Cultured leaf	ITS1 barcoding	HPLC-DAD	PCA	Brunakova et al.	2021 (138)
		ITS2 barcoding		HCA		
		Chromosome number			
		genome size				
**Sarsaparilla**	Dried root	rbcL barcoding	H-NMR	HCA	Kesanakurti et al.	2020 (82)
		matK barcoding				
		genome skimming				
		DNA probe				
***Glycyrrhiza*** **spp**.	Dried root powder	rbcL barcoding	H-NMR	PCA	Simmler et al.	2015 (139)
	Dried root stick	matK barcoding	UHPLC-UV	SIMCA		
	Dried root capsule	ITS barcoding		CDA		
		trnH-psbA barcoding			
***Echinacea*** **spp**.		Genome skimming	HPLC-UV		Handy et al.	2021 (140)
		metabaroding				
		matK barcoding				
		rbcL barcoding				

**Figure 13:**
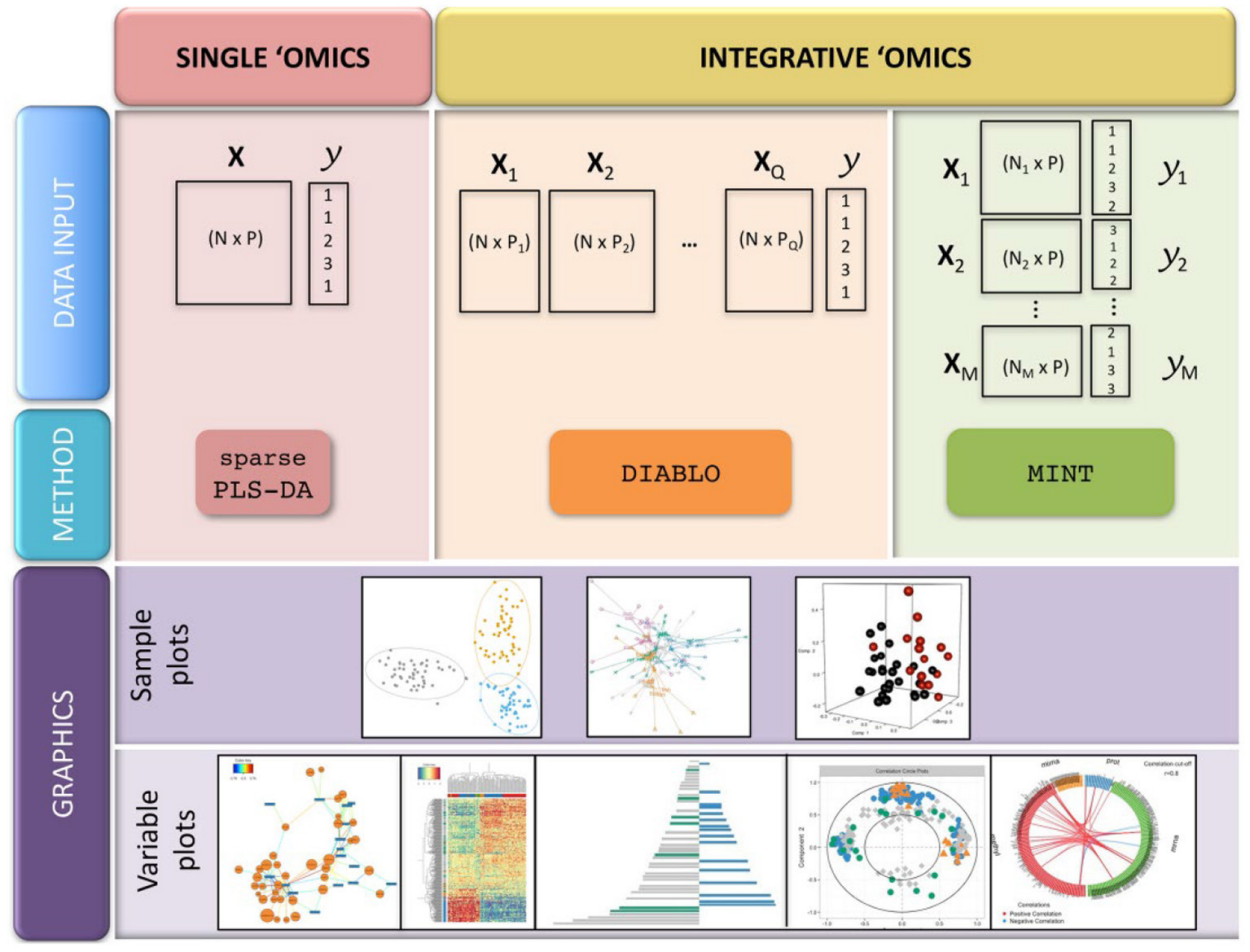
Integration of multiple-omics datasets and potential outputs using the mixOmics R package. Reproduced under a Creative Commons CC BY 4.0 license from Rohart et al. (151).

Although genetics is most integrated with genetics, there is potential to expand to lipidomics, proteomics, and transcriptomics. Lipidomics, the study of the complete set of lipids in an organism, is analytically similar to metabolomics; different extraction and analytical instrument methods target lipids. The same research lab could seamlessly transition from metabolite to lipid analysis since the instrumentation is often the same. On its own, lipidomics has been useful for detection of adulteration of white rice – RF and SVMs were used to discriminate pure and adulterated samples using LysoPCs and lysoPEs as novel lipid biomarkers (147). Using the same UPLC-MS instrument, Anagbogu et al. combined lipid and metabolite analysis to identify 30 genotypes of coffee; joining the two approaches increased species level resolution (148). Proteomics also uses similar instruments as metabolomics, but it has more variable methods that may complicate inter-lab experimentation. Peptide analysis allowed differentiation between mountain-cultivated ginseng and cultivated ginseng with 52 variable peptides between the groups (149), and MALDI TOF-TOF/MS yielded five proteins with potential to authenticate *Ophiocordyceps sinenis*, a traditional fungal medicine (150). Given the limited successful studies utilizing integrated -omics approaches for botanical product authentication and evidence that each approach has potential to identify adulteration, there is a gap in the botanical products community developing methods and statistical approaches for combined datasets. This is not a trivial undertaking; often the data sets generated for genomics, proteomics, and metabolomics experiments are very different, and their integration can be a challenge. The wide variety of expertise required to generate high quality data is also a factor in the wider implementation of a multi-omics approach to botanical authentication; these disparate techniques have different methodological proficiencies and even reagents and laboratory setups, necessitating broad proficiency in a single lab or a reliable collaboration between different laboratory groups. For data integration, the R tool mixOmics (including PCA, PLS, and PLS-DA tools) may prove useful for combined biomarker discovery and species identification (Figure 13) (151).

## When to Use What

**Figure 14:**
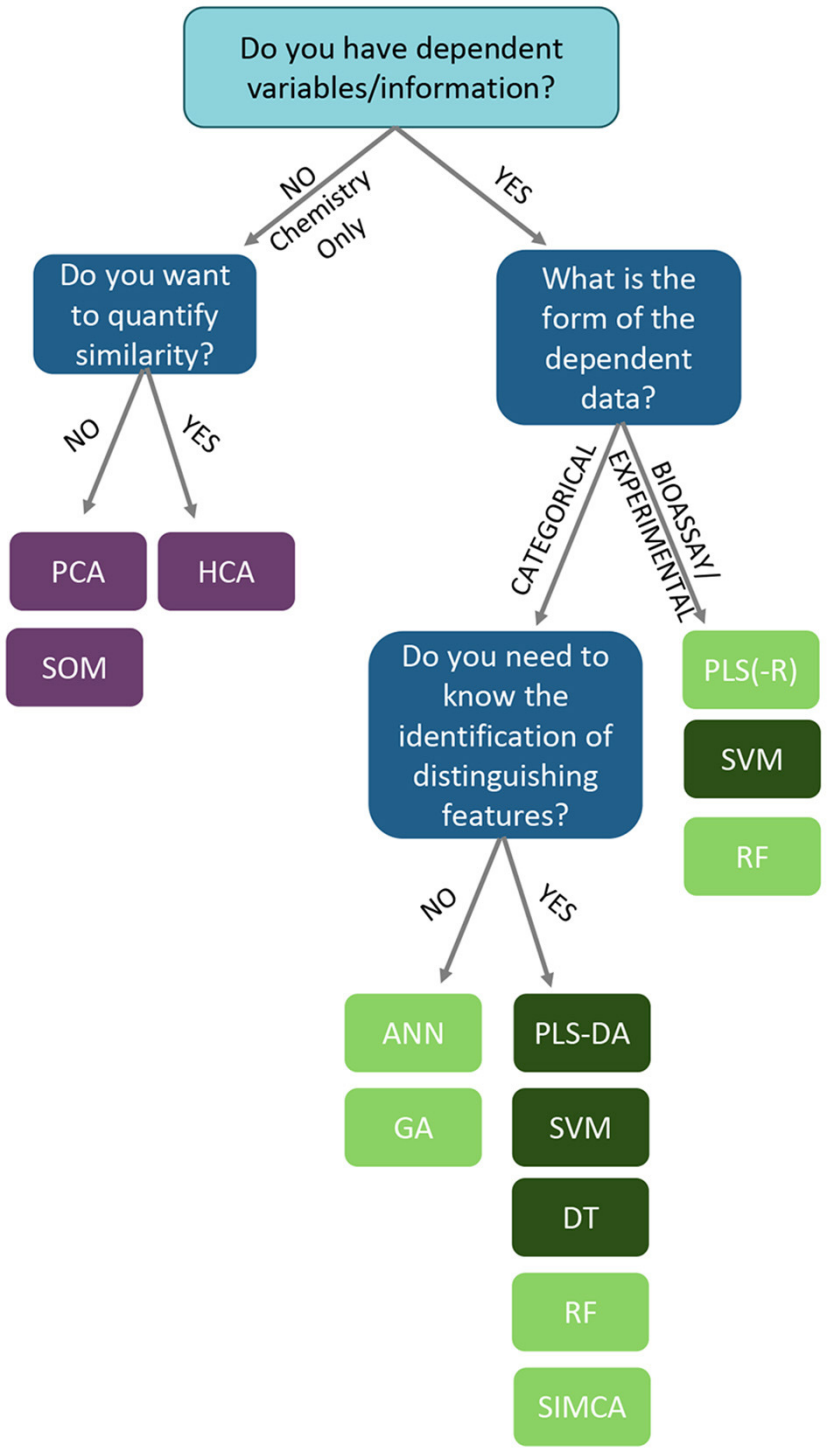
A decision tree to establish which analysis is more appropriate to analyzing complex chemical data. Based upon the presence of response data, and how the overall analysis needs to be structured/interpreted. ANN, artificial neural networks; DT, decision trees; GA, genomic algorithms; HCA, hierarchical clustering analysis; PCA, principal component analysis; PLS, partial least squares; PLS-DA, partial least squares-discriminate analysis; RF, random forests; SIMCA, soft independent modeling of class analogies; SOM, self-organized maps; SVM, support vector machines. Light green boxes represent “soft” classification techniques. Dark green boxes represent “hard” classification techniques.

This review highlights the number of chemometric techniques that can be applied to datasets in order to help authenticate botanical materials or detect adulteration. However, the diversity of approaches that are possible can be daunting to researchers unfamiliar with chemometric analysis and multivariate analysis/machine learning. While there is a bit of trial and error in selecting a chemometric approach, there are some points to consider in determining which technique to employ in analyzing a dataset. The decisions and chemometric options available to a researcher analyzing data are summarized in the following workflow (Figure 14).

First, is there response data collected with the chemical information? This could take the form of classification identifiers (e.g., “pure” vs. “adulterated”), control or QC datasets, taxonomic identification, quantitative data (e.g., temperature, geographic coordinates, elevation), or bioactivity data (inhibitory studies, cell studies, *in vivo* experiments, toxicological data, etc.). For datasets which do not contain any dependent information (only chemical input from FTIR, UV-VIS, MS, NMR, etc.), unsupervised analyses are recommended to understand the shape and relationships between samples without any guiding variables or observations. For a hierarchical analysis, where the similarity relationship between samples is ranked by distance, hierarchical cluster analysis (HCA) is the foremost choice. For examining similarities between samples without a hierarchy, principal component analysis (PCA), self-organizing maps (SOMs), and k-means clustering are viable options.

For experimental sets which contain dependent variables, chemometric options include numerous supervised analyses, which require response or dependent variables to train models. Generally, an unsupervised approach (PCA) should be applied to the metabolomics data set to ensure clustering occurs without predefined categories before delving into supervised analysis. Within the supervised approaches, the chemometric options vary depending on whether the response data are categorical or numerical in nature. Categorical dependent data, such as class assignments (e.g., “authentic” and “unknown”) enable supervised analysis to generate models that maximizes differences between the two classes. When choosing a classification methodology, one can consider whether the particular chemometric approach is “soft” or “hard.” These designations relate to a method's rigidness in assigning an unknown to a particular class. A “soft” classification rule estimates the probability associated with each class and subsequently provides a class prediction based on the largest estimated probability. In comparison, “hard” classification delivers a final class prediction without probabilistic reasoning behind the classification. Of the reviewed approaches, SIMCA, PLS, random forest (RF), genomic algorithms (GA), and artificial neural networks (ANN) are generally considered “soft” computational approaches (Figure 14, light green boxes) (152), while other techniques, such as PLS-DA, decision trees (DT), and support vector machines (SVM) (153) are “hard” methodologies (Figure 14, dark green boxes).

At this point, the last decision is the degree of interpretability the model will have for the researcher. A highly interpretable algorithm means that one can easily understand how any individual predictor (variable) is associated with the response, so it's easier to relate the final classification structure back to specific variables contributing to model responses. Techniques like partial least squares-discriminant analysis (PLS-DA), support vector machines (SVM), decision trees (DT), soft independent modeling of class analogies (SIMCA), and random forests (RF) are able to provide interpretable models. If interpretation is not essential (a “black box” approach), and only the final classification of the data is important, models like artificial neural networks (ANN) or genetic algorithms (GA)are prime options.

Numeric dependent variables are frequently obtained from biological activity experiments, and thus enable the use of prediction algorithms to correlate the dependent variable with variations in the chemical information. For the biochemometric analysis of these orthogonal datasets, partial least squares approaches (PLS, PLS-R) are most common in teasing out these relationships (92). However, newer machine learning approaches, such as SVM and RF, have the ability to provide predictive capabilities and understand relationships with input variables. As an example, Deng et al. employed random forests to provide geographical classification of green teas (which outperformed several other chemometric techniques), but also were able to correlate the geography with several isotopic indicators (103).

As with data-collection, where multiple orthogonal techniques facilitate a greater coverage of the overall chemical composition of the samples, multiple data analysis techniques are often utilized to gain a more comprehensive perspective of the data structure and relationship between samples. It is common to begin with unsupervised approaches (e.g., PCA) to glean a preliminary understanding of how samples are relating to one another, then followed up with supervised or machine learning methods to further classify the samples and obtain information about potential biomarkers or bioactive constituents. Zhang et al., in authenticating berry juices, first used PCA to identify clusters of juices by origin, then followed with PLS-DA to determine relevant biomarkers (76). PCA and HCA were employed to reveal well-differentiated clusters for black peppers, then followed by supervised PLS-DA for a prediction model for additional unknown samples (154). Thus, merging chemometric methods, when possible, offers researchers a potentially more rigorous analysis of their botanical data, which is essential to draw relevant and robust conclusions about authentication and adulteration questions.

## Conclusions and Future Directions

As the demand for botanical medicines and dietary supplements grows, in terms of relevance to human health as well as economic importance, ensuring reliable determination of starting materials for research, safety, and production considerations remains a challenge. Plant-based formulations pose a particularly unique hurdle due to their inherent chemical complexity as well as their variability. Non-targeted chemical fingerprinting techniques, including metabolomics, hold immense potential for describing the chemical composition of botanicals. However, organizing that highly complex information and deducing relevant conclusions from it can represent a major obstacle for researchers. This review has sought to address this hurdle by presenting examples of major chemometric techniques that can be employed to distill complex chemical data into models for authentication and classification of unknown samples. The adaptation of statistical models to wrangle large, complex datasets represents a significant advancement in modeling botanical chemical data. While the chemometric analysis methods profiled in this review are the most common, and some of the most powerful, approaches in use for botanical authentication, it is by no means an exhaustive list. Other variations of unsupervised and supervised techniques have been reported, and there is considerable research being undertaken to advance the capabilities of these statistical and machine learning approaches. And the combination of complementary methods (e.g., biological data and metabolomics, chemical profiling and genomics, or multi-omics techniques) has the potential to provide even more efficient and robust tools to advance authentication and discovery efforts.

## Author Contributions

JK and EA conceived, wrote and reviewed the manuscript, and secured funding. All authors contributed to the article and approved the submitted version.

## Funding

This work was supported in part by the USDA National Institute of Food and Agriculture and Hatch Appropriations (PEN04772), and the Garden Club of America's Anne S. Chatham Fellowship in Medicinal Botany.

## Conflict of Interest

The authors declare that the research was conducted in the absence of any commercial or financial relationships that could be construed as a potential conflict of interest.

## Publisher's Note

All claims expressed in this article are solely those of the authors and do not necessarily represent those of their affiliated organizations, or those of the publisher, the editors and the reviewers. Any product that may be evaluated in this article, or claim that may be made by its manufacturer, is not guaranteed or endorsed by the publisher.
